# Macrophage migration is differentially regulated by fibronectin and laminin through altered adhesion and myosin II localization

**DOI:** 10.1091/mbc.E23-04-0137

**Published:** 2024-01-12

**Authors:** Matthew W. Stinson, Sophia Liu, Alexander J. Laurenson, Jeremy D. Rotty

**Affiliations:** a Uniformed Services University of the Health Sciences, Department of Biochemistry, Bethesda, MD 20814; b The Henry M. Jackson Foundation for the Advancement of Military Medicine, Bethesda, MD 20817; Peking University Health Science Center

## Abstract

Macrophages are indispensable for proper immune surveillance and inflammatory regulation. They also exhibit dramatic phenotypic plasticity and are highly responsive to their local microenvironment, which includes the extracellular matrix (ECM). This work demonstrates that two fibrous ECM glycoproteins, fibronectin (FN) and laminin (LAM), elicit distinct morphological and migratory responses from macrophages in two-dimensional environments. LAM 111 inhibits macrophage cell spreading, but drives them to migrate rapidly and less persistently compared with cells on FN. Differential integrin engagement and ROCK/myosin II organization helps explain why macrophages alter their morphology and migration character on these two ECM components. This study also demonstrates that LAM 111 exerts a suppressive effect toward FN, as macrophages plated on a LAM/FN mixture adopt a morphology and migratory character almost identical to LAM alone. This suggests that distinct responses can be initiated downstream of receptor–ECM engagement, and that one component of the microenvironment may affect the cell’s ability to sense another. Overall, macrophages appear intrinsically poised to rapidly switch between distinct migratory characters based on their ECM environments. The role of ECM composition in dictating motile and inflammatory responses in three-dimensional and in vivo contexts warrants further study.

## Abstract (executive summary)

Cell migration has historically been studied on two-dimensional surfaces functionalized with extracellular matrix (ECM) components that allow cells to adhere and move efficiently. But how do different ECM components uniquely alter cellular structure and function?

Fibronectin spreads cells and stimulates slow, persistent migration. Laminin inhibits spreading and stimulates faster, less persistent migration. These ECMs stimulate signaling through distinct integrins that alter actomyosin organization, which in turn influences cell adhesion, structure, and dynamics.

These findings suggest that macrophage functions can be altered by their ECM microenvironment. Future studies should determine how ECM context alters macrophage function during wound healing and tumorigenesis.

## INTRODUCTION

Macrophages are innate immune cells that function in host defense, antigen presentation, and tissue repair (Italiani and Boraschi, 2014). Despite the importance of macrophages to these processes, it is not fully understood how each component of the local microenvironment contributes to the vast array of macrophage inflammatory phenotypes and functions (Murray and Wynn, 2011; Zhou *et al.*, 2014; Murray, 2017). Local cues can shift over time, leading macrophages to adopt an array of inflammatory or antiinflammatory profiles during physiological responses. Many disease states manifest with chronic inflammation, which may be due in part to dysregulated macrophage activation (Hao *et al.*, 2012; Yang and Ming, 2014; Buchacher *et al.*, 2015; Ma *et al.*, 2016; Schliefsteiner *et al.*, 2017). A better understanding of how the surrounding microenvironment alters macrophage function may be a key element of targeting dysregulated macrophage activation.

The extracellular matrix (ECM) is one such local cue that has been understudied from an immunomodulatory perspective compared with chemokines and cytokines. It has long been known that macrophages are primed by substrate adhesion (Haskill *et al.*, 1988; Yurochko *et al.*, 1992), and that the type of ECM glycoprotein present in their microenvironment can influence their activation. For example, fibronectin (FN) possesses an immunosuppressive character, while collagen (Col) promotes TNF-α and CSF induction (Eierman *et al.*, 1989). Vitronectin (VN) has more recently been found to stimulate IL-6 and LIF secretion (Keasey *et al.*, 2018). One especially compelling study noted that fibrinogen potentiates LPS/IFNγ-stimulated inflammation, while fibrin blocks it (Hsieh *et al.*, 2017). LAM induces MAPK signaling (Wei *et al.*, 1998) and was one of several ECMs reported to induce pro-inflammatory cytokines (Chana *et al.*, 2003). It is clear from the literature that these different ECM microenvironments are capable of differentially tuning macrophage behavior, but there is still much to learn about the mechanisms that drive these responses.

Integrins are major ECM-sensing heterodimeric transmembrane receptors that are ubiquitously expressed. Noncovalent interactions between α- (18 types) and β- (eight types) subunits form a total of at least 24 cell-type and tissue-dependent integrins. Active integrins must be stimulated either by intracellular signals, such as talin and kindlin binding to the cytoplasmic tail (called “inside-out” signaling), and/or by mechanical forces induced by the ECM itself (called “outside-in” signaling). These signals compete for access to adaptor and regulatory proteins, making it challenging to fully map their signaling pathways. A greater understanding of how these convergent and divergent signaling pathways respond to specific microenvironmental components is required in order to better understand physiological processes that occur in complex, mutable three-dimensional environments like those found in vivo.

This study reports that the ECM components FN and LAM can intrinsically alter the motility, morphology, and adhesion of macrophages through differential integrin activation leading to distinct nonmuscle myosin II organization. Using FN and LAM as model substrates for comparison, we have determined that ECM composition can alter macrophage migration. FN induces slow, relatively persistent migration, as well as spreading, elongation, and strong adhesion by stimulating integrin pairs like αvβ1, αvβ3, and α5β1 that bind to FN’s Arginine-Glycine-Aspartate (RGD) motif. Myosin IIA is predominantly localized at the bottom of the cell in this context. Conversely, LAM signaling through α6β1 integrin induces rapid, relatively random slingshot-like migration, a balled-up morphology and relatively weak adhesion. In this setting active myosin II is more evenly distributed throughout the cell. Taken together, these data suggest that local ECM context alone dramatically influences macrophage cell morphology and migration by signaling through specific integrins to alter myosin II subcellular localization, thereby allowing macrophages to immediately and dynamically respond to their environment.

## RESULTS

### Macrophage cell morphology and motility are tuned by the ECM

**FIGURE 1:**
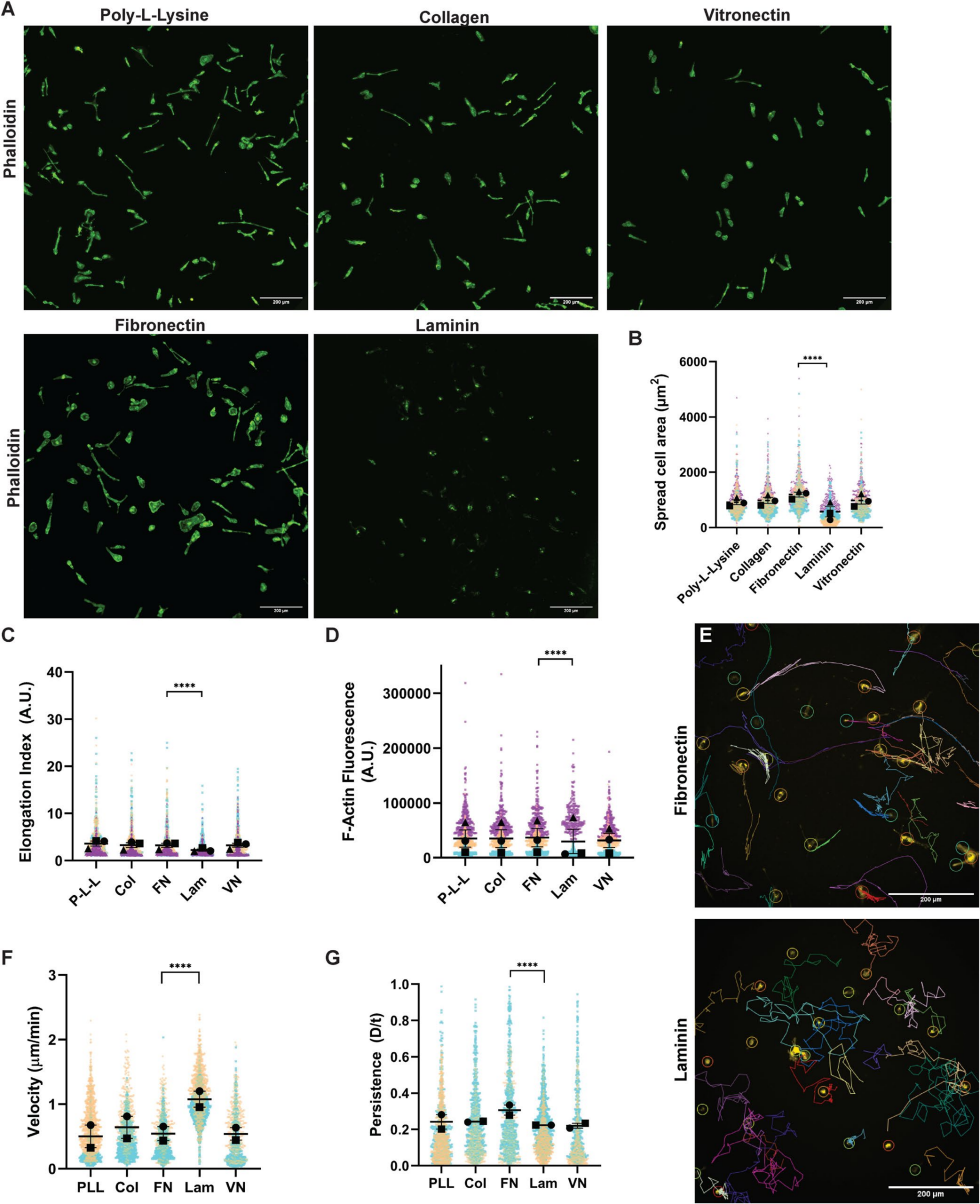
Macrophage cell morphology and motility are tuned by the ECM. (A) Representative images of fixed macrophages stained with Phalloidin 488 on 10 µg/ml P-L-L, Col, FN, LAM, or VN. Scale bar = 200 µm in each image. (B) Quantification of spread cell area in microns squared (****p < 0.0001, FN *n* = 967 cells, Lam *n* = 846 cells pooled from three independent experiments). Means and standard error of the mean for each experiment are represented with black symbols, all data points are plotted and each experimental run is color-coded. Statistical analysis was done by Kruskal–Wallis with Dunn multiple comparisons test. (C) Quantification of cellular elongation (A.U.). Greater elongation corresponds to a higher value in this analysis. (****p < 0.0001, FN *n* = 967 cells, Lam *n* = 846 cells pooled from the same three independent experiments used to generate data in Figure 1B). Means and standard error of the mean for each experiment are represented with black symbols, all data points are plotted and each experimental run is color-coded. Statistical analysis was Kruskal–Wallis with Dunn multiple comparisons test. (D) Total fluorescence of phalloidin staining (i.e., actin filaments) on each ECM. (****p < 0.0001, FN *n* = 967 cells, Lam *n* = 846 cells pooled from the same three independent experiments used to generate data in Figure 1, B and C). Means and standard error of the mean for each experiment are represented with black symbols, all data points are plotted and each experimental run is color-coded. Statistical analysis was done by Kruskal–Wallis with Dunn multiple comparisons test. (E) Representative tracks of macrophages migrating randomly on 10 µg/ml FN or LAM over a 16 h time period, generated by the TrackMate FIJI plugin. Scale bar = 200 µm. (F) Velocity (cell speed) in microns per minute and (G) Persistence (d/T) of randomly migrating macrophages on 10 µg/ml the indicated ECM (****p < 0.0001, ***p = 0.0001, P-L-L *n* = 1264 tracks, Col *n* = 959 tracks, FN *n* = 1004 tracks, Lam *n* = 1559 tracks, VN *n* = 773 tracks pooled from two experiments). Means and standard error of the mean for each experiment are represented with black symbols, all data points are plotted and each experimental run is color-coded. Statistical analysis was done by Kruskal–Wallis with Dunn multiple comparisons test.

We first plated primary bone marrow–derived murine macrophages (BMDM) on equal concentrations of various ECMs to test the hypothesis that the matrix microenvironment influences cell morphology and cytoskeletal organization. Macrophages produced F-actin at high levels and spread well in many ECM contexts (Figure 1A), except when exposed to LAM 111. LAM uniquely impaired cell spreading, caused macrophages to adopt a less elongated morphology, and suppressed F-actin polymerization (Figure 1, A–D). Conversely, FN strongly stimulated cell spreading compared with the other ECMs (Figure 1, A and B), and supported cellular elongation and F-actin polymerization (Figure 1, C and D). Statistical relationships between all ECMs tested can be found in Supplemental Figure 1. These data indicate that the local ECM environment is sufficient to alter cell morphology and the actin cytoskeleton.

Given the cytoskeleton’s role in dynamic cellular processes we hypothesized further that ECM environment influences cell motility. LAM and FN were chosen as model systems to further interrogate this idea, as macrophages were significantly altered by interaction with these ECMs. Macrophages plated on FN adopted a more mesenchymal-like behavior during random migration experiments, moving via coherent leading edge protrusion coupled to retraction at the cell rear (Supplemental Movie 1, left). On the other hand, LAM drove macrophages to adopt a chaotic style of migration during random migration experiments that largely dispenses with stable edge protrusion in favor of a more randomized, shorter lived protrusion cycle (Supplemental Movie 1, right). As a result, migration on FN gave rise to linear migration tracks, while LAM induced meandering tracks characterized by frequent directional changes (Figure 1E). As a result, LAM-plated cells migrated faster, but changed direction more often (i.e., they are less directionally persistent; Figure 1, F and G). Conversely, macrophages on FN were relatively slow and were less likely to change direction (Figure 1, F and G). This result was confirmed with serum-free macrophage media, ruling out the interference of undefined components in bovine serum that alter motility independently of the plated ECMs (Supplemental Figure 1, A and B). Though macrophages demonstrated motility and morphology differences on Col, VN, and poly-l-lysine (P-L-L), these conditions elicited mesenchymal-like motility similarly to FN (Figure 1, F and G; Supplemental Figure S1C). These data together demonstrate that macrophage morphology, cytoskeletal regulation, and motility are inherently tunable by the ECM microenvironment.

**Figure:** Movie S1 WT macrophages, random migration on 10 μg/mL fibronectin (left) and 10 μg/mL laminin (right).

### Macrophage response to ECM composition is concentration and integrin-dependent

**FIGURE 2:**
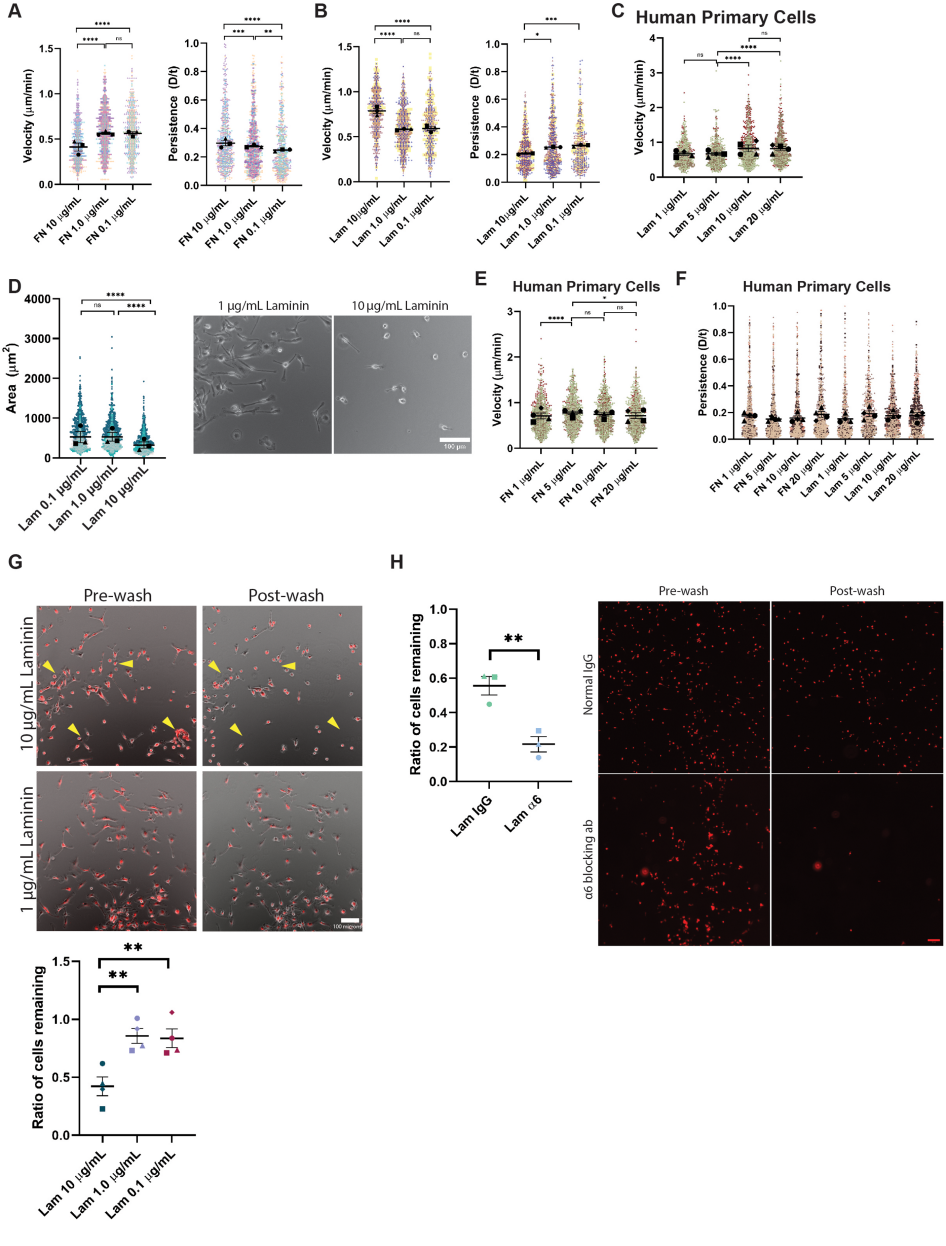
Macrophage response to ECM composition is concentration and integrin-dependent. (A) Velocity (cell speed) in microns per minute and persistence (d/T) for macrophages migrating randomly on the indicated concentration of FN. Means and standard error of the mean for each experiment are represented with black symbols, all data points are plotted and each experimental run is color-coded. Statistical analysis was done by Kruskal–Wallis with Dunn multiple comparisons test. ****p < 0.0001, ***p = 0.0004, **p = 0.0022, ns = not significant. FN 10 μg/ml *n* = 701 tracks, 1.0 μg/ml *n* = 840 tracks, 0.1 μg/ml *n* = 656 tracks. These data were pooled from three independent experiments. (B) Velocity (cell speed) in microns per minute and persistence (d/T) for macrophages migrating randomly on the indicated concentration of LAM. Means and standard error of the mean for each experiment are represented with black symbols, all data points are plotted and each experimental run is color-coded. Statistical analysis was done by Kruskal–Wallis with Dunn multiple comparisons test. ****p < 0.0001, ***p = 0.0002, *p = 0.0182, ns = not significant. Lam 10 μg/ml *n* = 590 tracks, 1.0 μg/ml *n* = 651 tracks, 0.1 μg/ml *n* = 454 tracks. (C) Velocity (cell speed) in microns per minute for human primary macrophages migrating randomly on the indicated concentration of LAM. Means and standard error of the mean for each experiment are represented with black symbols, all data points are plotted and each experimental run is color-coded. Statistical analysis was done by Kruskal–Wallis with Dunn multiple comparisons test. **** *p* < 0.0001. *n* = at least 688 cell tracks pooled from four independent experiments. (D) Spread cell area in microns squared (Left), for macrophages plated on the indicated concentration of LAM. Means and standard error of the mean for each experiment are represented with black symbols, all data points are plotted and each experimental run is color-coded. Statistical analysis was Kruskal-Wallis with Dunn multiple comparisons test. ****p < 0.0001. Lam 0.1 μg/ml *n* = 1439 cells, Lam 1.0 μg/ml *n* = 1293 cells, Lam 10 μg/ml *n* = 1279 cells pooled from three experiments. Representative images of 1.0 μg/ml and 10 μg/ml are shown (*Right*) for comparison. Scale bar = 100 µm. Uncropped versions of these images can be found in Supplemental Figure 4. (E) Velocity (cell speed) in microns per minute for human primary macrophages migrating randomly on the indicated concentration of FN. Means and standard error of the mean for each experiment are represented with black symbols, all data points are plotted and each experimental run is color coded. Statistical analysis was done by Kruskal–Wallis with Dunn multiple comparisons test. ****p < 0.0001, *p = 0.0126. *n* = at least 967 cell tracks pooled from four independent experiments. (F) Persistence (d/T) for human primary macrophages migrating randomly on the indicated concentration of FN or LAM. Means and standard error of the mean for each experiment are represented with black symbols, all data points are plotted and each experimental run is color coded. *n* = at least 688 cell tracks per condition, the same tracks used to quantify velocity in panels (C) and (E), pooled from four independent experiments. (G) Adhesion assay for macrophages plated on the indicated concentration of LAM. Cell number was counted for each field of view before and after gentle washing. Data represents the ratio of total number of bound cells postwash divided by the total number of cells prewash for each condition. Statistical analysis was done with one-way ANOVA with multiple comparisons test. Lam 10 and Lam 1.0 μg/ml **p = 0.0069, LAM 10 and LAM 0.1 μg/ml **p = 0.0092, *N* = 4 independent experiments. Right: Representative images of tracker dye labeling superimposed on phase contrast images during indicated condition. Yellow arrowheads indicate regions where cells are present in the prewash condition and missing after gentle washing. Uncropped versions of these images can be found in Supplemental Figure S4. Scale bar = 100 µm. (H) Macrophages plated on 10 μg/ml LAM after 1 h incubation at 37°C with either IgG isotype control antibody or α6 blocking antibody. Data was analyzed and quantified identically to adhesion assay data in panel 2D. However, in this case an unpaired *t* test was used to determine statistical significance, **p = 0.0084, *N* = 3 independent experiments. Right: Representative images of tracker dye labeling pre- and postwashing after exposure to IgG control or α6 blocking antibody. Scale bar = 100 µm.

Macrophages display fundamentally different responses to FN and LAM. We, therefore, wondered whether varying the amount or relative composition of ECM in the microenvironment would also alter macrophage cellular dynamics. Macrophages moved significantly faster and less persistently on lower FN concentrations than cells on higher concentrations (Figure 2A; Supplemental Movie 2, left). Macrophages demonstrated a similar trend on the same concentration range of Col and VN (Supplemental Figure S2, A and B). These data indicate an inverse correlation between adhesion strength and migration speed, a fundamental relationship that has been previously described (DiMilla *et al.*, 1993; Wu *et al.*, 2012). Many ECM receptors exist on the surface of macrophages, with the heterodimeric integrins being chief mediators of adhesion. Perhaps high levels of integrin engagement with FN reinforce leading edge maintenance and directional persistence at the expense of cell speed. In contrast, macrophages plated on a relatively high concentration of LAM were faster and less persistent than their counterparts plated on lower LAM concentrations (Figure 2B; Supplemental Movie 2, right). It is also worth noting the inverse relationship between cell speed and migratory persistence in these random migration experiments, similar to Figure 1, F and G. We decided to test whether human cells responded similarly to mouse cells across LAM and FN gradients. Experiments conducted with human cells are explicitly indicated as such, while experiments in mouse macrophages will often be referred to as simply “macrophages”. Human primary monocyte-derived macrophages (MDMs) also migrated faster on higher concentrations of LAM (Figure 2C) and changed shape dramatically, becoming smaller and more circular as LAM concentration increases (Supplemental Figure 2, C and D), similar to findings in mouse primary macrophages (Figures 1, B and C, 2D). Primary human macrophages on FN showed a modest, but significant, motility shift reminiscent of a biphasic response (Figure 2E), similar to findings with mouse primary macrophages. However, human primary macrophages did not show the same persistence trend as mouse primary cells on either ECM (Figure 2F). Previous studies have advanced the idea that macrophages do not adhere well to LAM despite expressing the LAM-binding α6β1 integrin (Mercurio and Shaw, 1988; Shaw and Mercurio, 1989). To test whether substrate adhesion plays a role in the LAM response, we allowed cells to spread on different concentrations of LAM and quantified how many remained after gentle washing. Mouse macrophages on lower LAM concentrations spread and adhered relatively well (Figure 2G), arguing that the failure to spread and adhere on 10 µg/ml LAM is a deliberate response to sensing relatively high levels of LAM. Human macrophages also tended to demonstrate lower adhesion on higher concentrations of LAM, even when assayed 48 h after plating on ECM-coated surfaces (Supplemental Figure S2E). This is echoed by previous reports that have seen a similar trend (Mercurio and Shaw, 1988). Mouse macrophages also adhered well to low LAM concentrations in serum free media with and without bovine serum albumin (BSA) backfill (Supplemental Figure S2F). This finding argues that the adhesive effect seen on low LAM concentrations in Figure 2E is not due to nonspecific coating of glass by serum factors present in the media. We confirmed this by treating LAM-coated coverslips (± BSA backfill) with serum-containing macrophage media. These coverslips were poorly coated compared with coverslips coated with purified FN (10 mM, Supplemental Figure S2G), with only slightly more FN antibody signal than completely uncoated glass (no ECM SFM, Supplemental Figure S2G). Our data supports the notion that high concentrations of LAM do affect macrophage adhesion. However, blocking α6 integrin via neutralizing antibody lowered adhesion to 10 µg/ml LAM even further than baseline (Figure 2H), demonstrating that integrin-based adhesion does occur in this setting. Deposition of fluorescent LAM and FN across these concentration ranges was confirmed, arguing that the differences seen here are not due to defective LAM binding to our glass surfaces (Supplemental Figure S2H). In total, these studies indicate that macrophages use a different style of integrin-based adhesion to bind to LAM, which facilitates a distinct cellular response compared with RGD-based FN adhesion.

**Figure:** Movie S2 WT macrophages, random migration on 1 μg/mL fibronectin (left) and 1 μg/mL laminin (right).

### Cells change shape dramatically and “slingshot” on LAM

**FIGURE 3:**
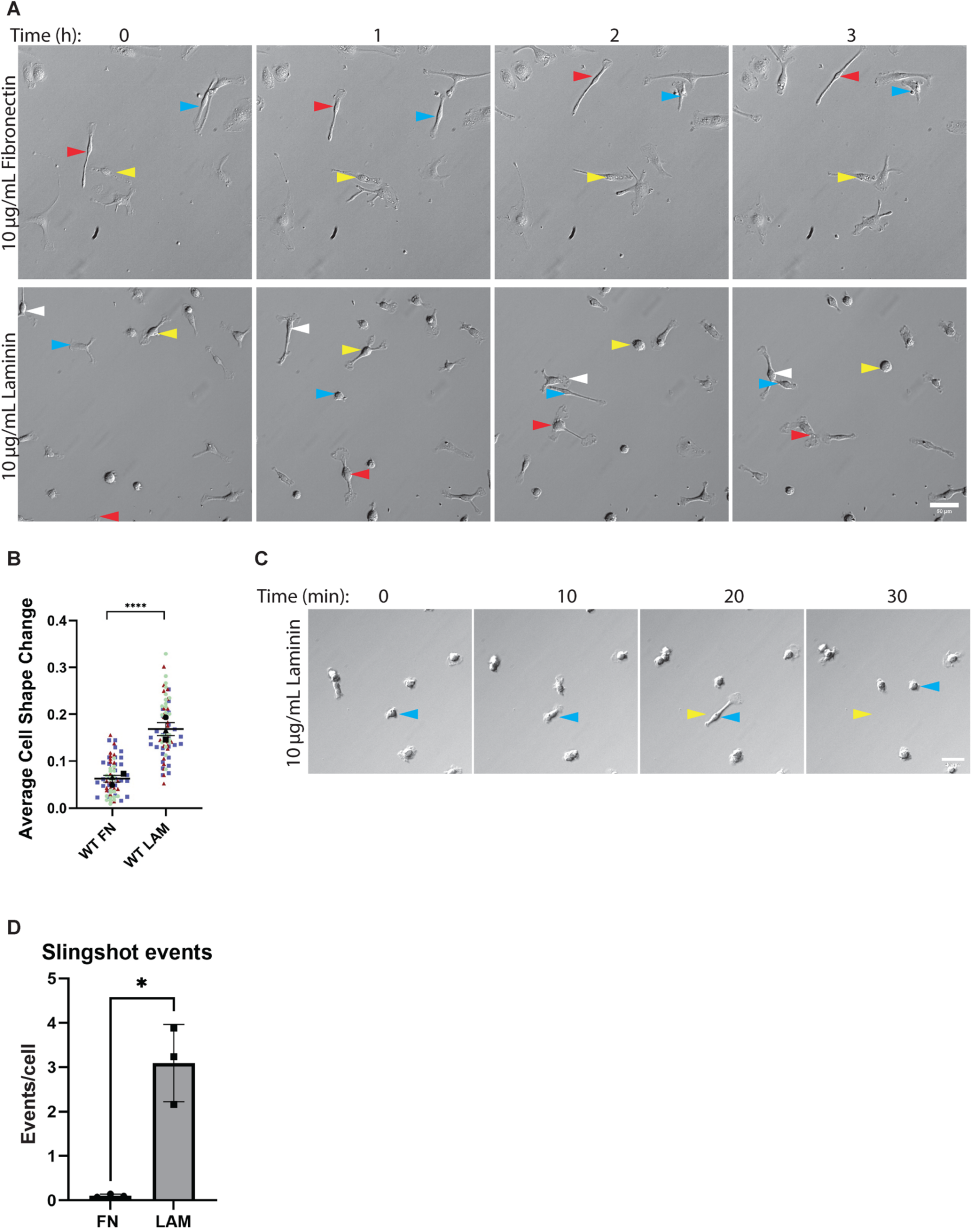
FN and LAM induce distinct motility behaviors. (A) Representative relief contrast images demonstrating random migration modes of macrophages on LAM and FN. Blue, red, and yellow arrowheads in relief contrast images mark different individual cells over the 3-h period. This image series is part of a longer time lapse experiment. The unabridged, uncropped data can be found in Supplemental Movies 1 and 2. Images in this panel correspond to frames 1, 7, 13, and 19 of the respective movie. Scale bar = 50 µm. (B) Changes in cellular circularity occurring from frame to frame (10-min intervals) during random motility over the course of 5 h. Means of each experiment and standard error of the mean are represented with black symbols, all data points are plotted, and each experimental run is color-coded. ****p < 0.0001, analyzed via Mann–Whitney test. FN *n* = 77 cells, LAM *n* = 75 cells. These data were pooled from three independent experiments. (C) Representative image of a macrophage undergoing a slingshot event after plating on LAM. Blue arrowheads mark the position of the cell body in each frame. Yellow arrowheads correspond to the original position of the cell body just before a slingshot event. Scale bar = 40 µm. (D) Number of slingshot events per cell in each condition, when analyzed during the 5-h intervals used to generate data in Figure 3B. A slingshot event was defined as a change in cell shape ≥ 0.4 from one frame to the next. Events/cell was determined for each experimental run, and are plotted here with the standard error of the mean. These data were analyzed with an unpaired *t* test using Welch correction. *p = 0.0269. *N* = 3 experiments.

To better understand the effects of LAM and FN on macrophage cellular dynamics, we analyzed the frame-to-frame cell shape changes of macrophages migrating on LAM and FN. Macrophages plated on LAM were highly dynamic and changed morphology often, while their counterparts on FN maintained a front-rear polarity that made it possible to migrate in straight lines for hours at a time (Figure 3A, arrowheads). The dynamic nature of the LAM-plated cells is quantifiable, as their ability to transition rapidly between balled up (more circular) and elongated (less circular) morphologies from one frame to the next was substantially higher than macrophages plated on FN (Figure 3B). Macrophages migrating on LAM often skated around as they randomly protrude and change direction often, as in Figure 3A. However, these cells can also undergo a process on LAM that we refer to as “slingshot motility” or “slingshotting” in which macrophages elongated before releasing the cell rear and launching the cell body forward (Figure 3C). While slingshot motility is not the only strategy macrophages use to migrate on LAM, it does appear to be uniquely prevalent on LAM and to happen rarely on FN (Figure 3D). Together, these data reveal that macrophage motility on LAM is characterized by rapid and dynamic cell shape changes that give rise to their meandering migration style, whereas macrophages on FN have a stable shape that maintains directional persistence.

### Nonmuscle myosin II drives the rapid cell shape changes that contribute to fast motility on LAM

**FIGURE 4:**
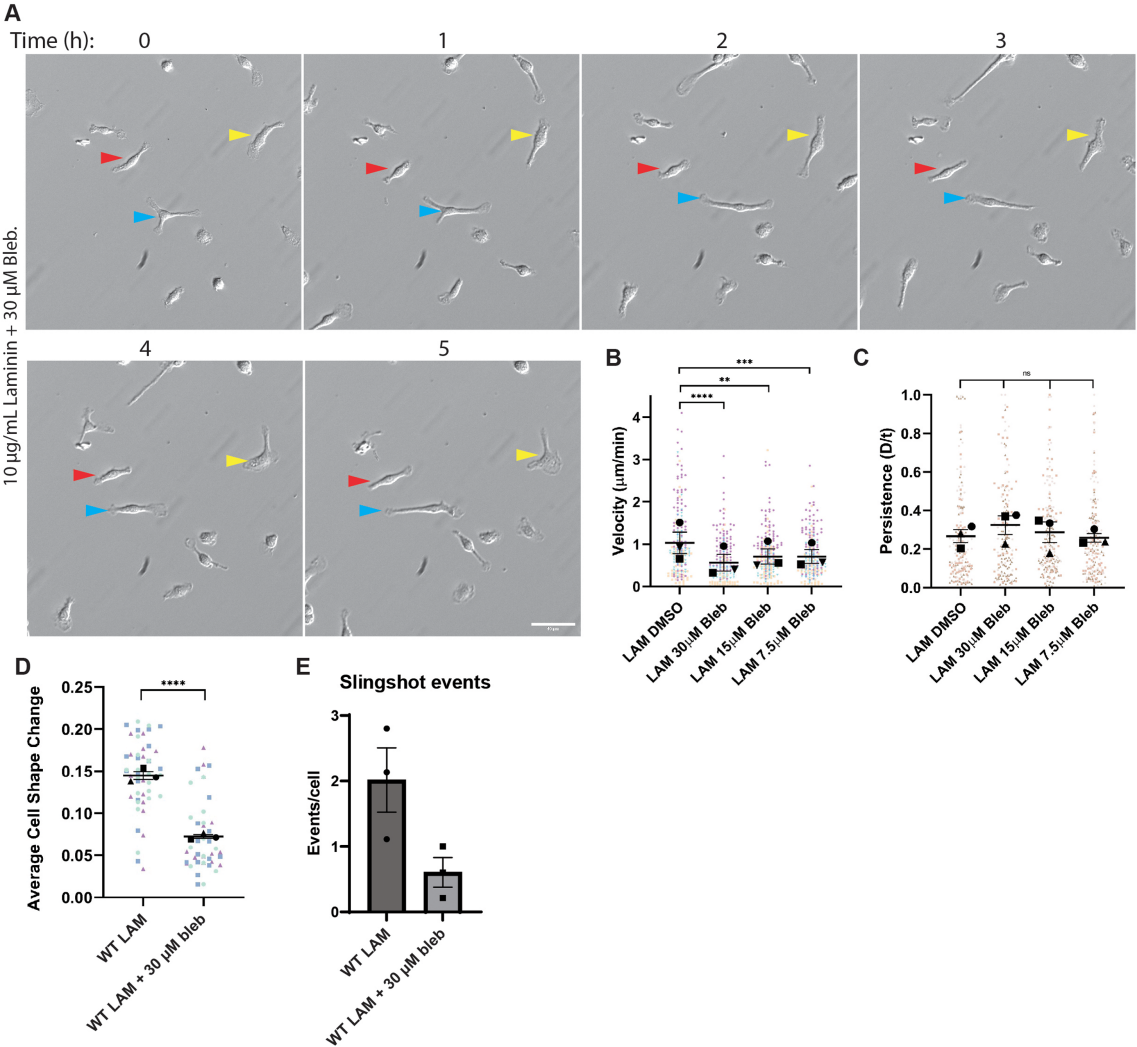
Nonmuscle myosin II drives the rapid cell shape changes that contribute to fast motility on LAM. (A) Representative relief contrast images demonstrating random migration of LAM-plated macrophages migrating in the presence of 30 μM blebbistatin, which inhibits nonmuscle myosin II. Blue, red, and yellow arrowheads in relief contrast images mark different individual cells over the 5-h period. This image series is part of a longer time lapse experiment. The unabridged, uncropped data can be found in Supplemental Movie 5. Images in this panel correspond to frames 1, 7, 13, 19, 25, and 31 of the movie. Scale bar = 50 µm. (B) Velocity (cell speed) in microns per minute and (C) persistence (d/T) for macrophages migrating on LAM and subjected to indicated doses of blebbistatin, or DMSO as the negative control. Means and standard error of the mean for each experiment are represented with black symbols, all data points are plotted and each experimental run is color-coded. Statistical analysis was Statistical analysis was done by Kruskal–Wallis with Dunn multiple comparisons test. ****p < 0.0001, ***p = 0.0004, **p = 0.0031, ns = not significant; Lam DMSO *n* = 200 tracks, Lam 30 μM Blebbistatin *n* = 172 tracks, Lam 15 μM Blebbistatin *n* = 204 tracks, Lam 7.5 μM Blebbistatin *n* = 197 tracks pooled from three experiments). (D) Changes in cellular circularity occurring from frame to frame (10-min intervals) during random motility over the course of 5 h. Means of each experiment and standard error of the mean are represented with black symbols, and all data points are plotted and each experimental run is color-coded. ****p < 0.0001, analyzed via Mann–Whitney test, Lam *n* = 48 cells, Lam 30 μM Blebbistatin *n* = 44 cells pooled from three independent experiments. (E) Number of slingshot events per cell in each condition, when analyzed during the 5-h intervals used to generate data in panel (D). A slingshot event was defined as a change in cell shape ≥ 0.4 from one frame to the next. Events/cell was determined for each experimental run, and are plotted here with the standard error of the mean.

Several key phenotypic elements of macrophage motility on LAM resembled amoeboid motility. Their lack of spreading coupled with rapid, meandering migration suggested that these cells were not moving exclusively via lamellipodial protrusions. Rather, macrophages on LAM seemed to be using cellular contractility to skate and slingshot over the surface instead of crawling. These observations led us to test whether myosin II contractility contributed to the LAM phenotype. LAM-plated macrophages treated with the nonmuscle myosin II inhibitor S-nitro-blebbistatin (hereafter referred to as blebbistatin) were less dynamic than control cells (Figure 4A; Supplemental Movie 3). All tested concentrations of blebbistatin decreased macrophage migration speed on LAM, but had no effect on persistence (Figure 4, B and C). Interestingly, blebbistatin also blocked dynamic cell shape changes and suppressed slingshot motility (Figure 4, D and E). This finding is highly reminiscent of the myosin-dependent slingshot motility seen when mesenchymal cells migrate in deformable three-dimensional matrices (Wang *et al.*, 2019). Macrophages plated on FN were less severely affected by blebbistatin, with only the highest blebbistatin dose slowing motility and no effect on persistence at any dose (Supplemental Figure S3, A and B). These data indicate that myosin II activity is a fundamental contributor to the dynamic cell shape changes that drive rapid macrophage motility on LAM.

**Figure:** Movie S3 WT macrophages, random migration on 10 μg/mL laminin + 30 μM blebbistatin.

### Dual inhibition of myosin II and ROCK suppresses the LAM phenotype

**FIGURE 5:**
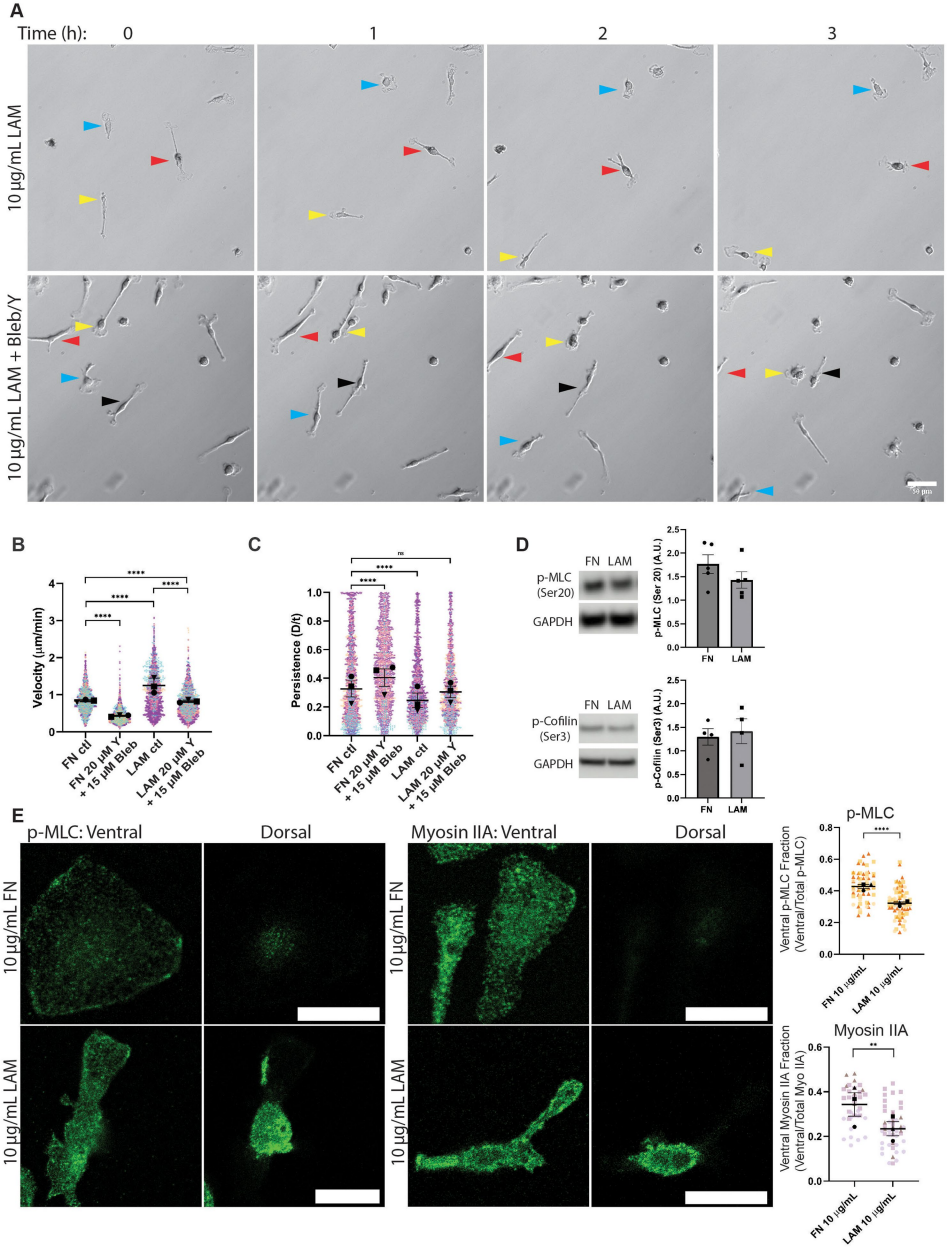
Dual inhibition of myosin II and ROCK suppresses LAM phenotype. (A) Representative relief contrast images demonstrating random migration of LAM-plated macrophages treated with DMSO or a combination of 20 μM Y-27632 (ROCK inhibitor, “Y”) and 15 μM Blebbistatin (“Bleb”). Blue, red, and yellow arrowheads in relief contrast images mark different individual cells over the 3-h period. These are part of a longer time lapse experiment. The unabridged, uncropped images of the dual inhibitor condition can be found in Supplemental Movie 6. Images in this figure correspond to frames 25, 31, 37, and 43 of Supplemental Movie 6. Scale bar = 50 µm. (B) Velocity (cell speed) in microns per minute and (C) persistence (d/T) for macrophages migrating on FN or LAM and subjected to DMSO (ctl) or 20 μM Y-27632 (“Y”) + 15 μM Blebbistatin (“Bleb”). Means and standard error of the mean for each experiment are represented with black symbols, all data points are plotted and each experimental run is color-coded. Statistical analysis was Kruskal−Wallis with Dunn multiple comparisons test. ****p < 0.0001, ***p = 0.0005, ns = not significant; FN ctrl *n* = 1826 tracks, FN 20 μM Y-27632 15 μM Bleb *n* = 1779 tracks, Lam ctrl *n* = 1712 tracks, Lam FN 20 μM Y-27632 15 μM Bleb *n* = 1539 tracks pooled from three experiments. (D) Left: Western blot images of p-MLC (Ser20), and p-cofilin (Ser3). GAPDH is included as a loading control. *Right*: Quantification of p-MLC and p-cofilin signal after normalization to GAPDH. *N* = 5 for p-MLC and *N* = 4 for p-cofilin. Bars represent standard error of the mean. Uncropped images of these blots can be found in Supplemental Figure 5. (E) Left: Representative images of p-MLC and myosin IIA staining in macrophages plated on FN or LAM. All scale bars are 25 microns. *Right*: Quantification of myosin IIA and p-MLC fluorescence level in the lowest confocal z-slice relative to the total amount of fluorescence staining in the cell. Means and standard error of the mean for each experiment are represented with black symbols, all data points are plotted and each experimental run is color-coded. Statistical analysis was evaluated with an unpaired *t* test. **p value = 0.0054, ****p value < 0.0001. *n* = 37 cells for myosin II on FN, 49 cells for myosin II on LAM, 66 cells for p-MLC on FN, and 78 cells for p-MLC on LAM, pooled from three experiments.

Rho-associated protein kinase (ROCK) plays a fundamental role in modulating the actin cytoskeleton (Maekawa *et al.*, 1999). Though it is a major activator of myosin contractility (Kimura *et al.*, 1996), ROCK also influences the activity of cofilin (Maekawa *et al.*, 1999), Ezrin/Radixin/Moesin (ERM) proteins (Matsui *et al.*, 1998; Hebert *et al.*, 2008), and the formin FHOD1 (Takeya *et al.*, 2008). All of these proteins have been implicated in dynamic cellular processes like motility. Because myosin inhibition alone did not raise migratory persistence on LAM, we wondered whether dual inhibition with blebbistatin and the ROCK inhibitor Y-27632 would cause macrophages on LAM to behave more like cells plated on FN. Macrophage cell speed on LAM was significantly reduced by dual inhibition of myosin II and ROCK, to the extent that they were slightly slower than untreated macrophages on FN (Figure 5, A and B; Supplemental Movie S4, right). Dual inhibition also raised migratory persistence on LAM, to the extent that this population became indistinguishable from the FN control cells (Figure 5C; Supplemental Movie 4, right). Macrophages plated on FN were also slower and more persistent with dual inhibition compared with untreated cells on FN (Figure 5, B and C; Supplemental Movie 4, left). All of these data indicate that myosin II and ROCK activity are crucial to the LAM phenotype. We hypothesized that this effect is due to LAM’s ability to activate ROCK and myosin contractility more efficiently than FN. ROCK activation leads to phosphorylation of myosin regulatory light chain (MLC), leading to activation of myosin II contractility (Kureishi *et al*., 1997; Kawano *et al.*, 1999). Additionally, ROCK activates LIM kinase (Maekawa *et al.*, 1999), which in turn phosphorylates cofilin, thereby inactivating it (Arber *et al.*, 1998; Yang *et al.*, 1998). Therefore, to interrogate our hypothesis further, p-MLC (Ser20) and p-cofilin (Ser3) were used as readouts for ROCK activity on FN and LAM. Contrary to our expectation, phosphorylation of MLC at Ser20 and cofilin at Ser3 were not significantly different when cells were plated on the two ECMs, when normalized to GAPDH (Figure 5D). We then wondered whether the activation levels of myosin and ROCK were less important than how the two proteins localized within macrophages when interacting with each ECM component. We used myosin IIA and p-myosin light chain (p-MLC) staining in fixed cells imaged via confocal to gain quantitative insight into myosin localization on FN and LAM. A greater fraction of the total myosin IIA and p-MLC signal was present at the bottom (ventral surface) of FN-plated cells (Figure 5E), reflecting an alteration in active myosin localization toward the adhesive plane in these cells. Though myosin II and ROCK clearly contribute to the LAM phenotype, we find no evidence for hyperactivation of contractile signaling. Instead, the contractile apparatus that is normally in close proximity to the integrin-containing bottom of the cell (on FN) is redistributed on LAM and appears to be spread more evenly throughout the cell.

**Figure:** Movie S4 WT macrophages, random migration on 10 μg/mL fibronectin + 20 μM Y‐27632 and 15 μM blebbistatin (left) and 10 μg/mL laminin + 20 μM Y‐27632 and 15 μM blebbistatin (right).

### Macrophages preferentially sense LAM in mixed ECM conditions

**FIGURE 6:**
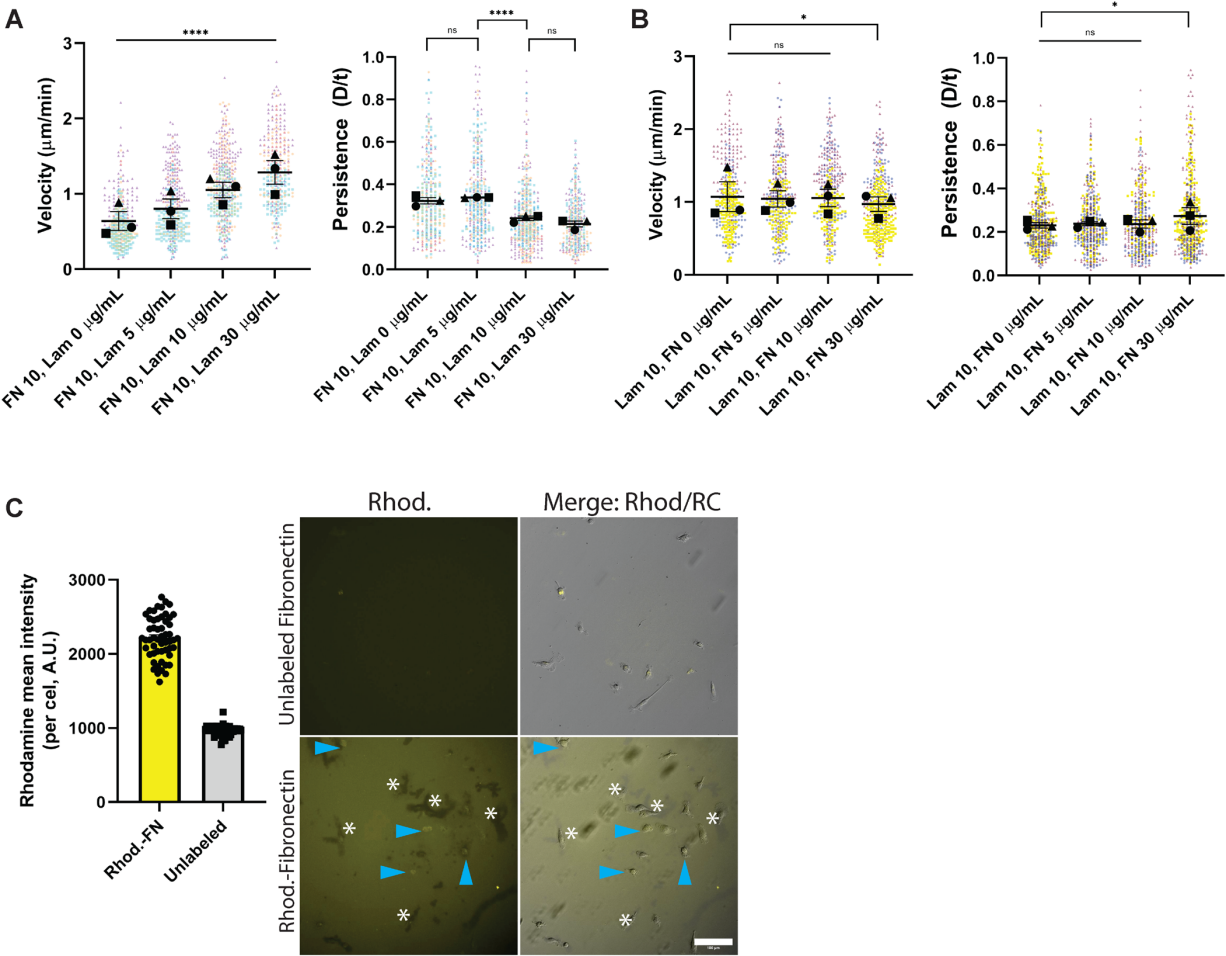
Macrophages preferentially sense LAM in mixed ECM conditions. (A) Velocity (cell speed) in microns per minute and persistence (d/T) for macrophages migrating on FN or FN + LAM at various concentrations. Means and standard error of the mean for each experiment are represented with black symbols, all data points are plotted and each experimental run is color-coded. Statistical analysis was Kruskal−Wallis with Dunn multiple comparisons test. ****p < 0.0001, ns = not significant; FN 10 Lam 0 μg/ml *n* = 360 tracks, FN 10 Lam 5 μg/ml *n* = 374 tracks, FN 10 Lam 10 μg/ml *n* = 460 tracks, FN 10 Lam 30 μg/ml *n* = 335 tracks, pooled from three experiments. (B) Velocity (cell speed) in microns per minute and persistence (d/T) for macrophages migrating on LAM or LAM + FN at various concentrations. Means and standard error of the mean for each experiment are represented with black symbols, all data points are plotted and each experimental run is color-coded. Statistical analysis was Kruskal−Wallis with Dunn multiple comparisons test. *p = 0.0124, for persistence, *p = 0.0273, for velocity; Lam 10 FN 0 μg/ml *n* = 424 tracks, Lam 10 FN 5 μg/ml *n* = 393 tracks, Lam 10 FN 10 μg/ml *n* = 394 tracks, Lam 10 FN 30 μg/ml *n* = 456 tracks, pooled from three experiments. Examples of cells migrating on mixed ECMs can be found in Supplemental Movies 8–10. (C) *Left*: Rhodamine fluorescence intensity of macrophages plated on Rhodamine-FN or unlabeled FN. Right: Representative images of Rhodamine intensity, alongside Rhodamine/relief contrast merge. Blue arrowheads denote macrophages that are Rhodamine+, while white asterisks highlight areas where FN appears to have been ripped away. Scale bar = 100 µm. Uncropped versions of these images can be found in Supplemental Figure 4.

Macrophages respond to a LAM-rich environment by utilizing myosin contractility to establish a rapid, meandering mode of migration. We next sought to determine whether this response was a passive one arising from an inability to adhere well to LAM, or a deliberate response to a LAM-rich environment. FN induces strong adhesion and spreading in macrophages, so its inclusion alongside LAM should suppress the LAM phenotype if the latter arises from impaired adhesion. In contrast to this expectation, the migratory characteristics of macrophages in the mixed ECM context was largely influenced by how much LAM was present. Macrophages migrated faster and less persistently in a LAM dose-dependent manner when FN is held at our baseline concentration of 10 µg/ml (Figure 6A; Supplemental Movie 5, left and middle). On the other hand, when LAM is held constant at 10 µg/ml, both migration speed and persistence were largely resistant to increasing concentrations of FN (Figure 6B). At 10 µg/ml LAM + 30 µg/ml FN macrophages slowed slightly in response to FN, but their motility was still higher than baseline FN migration (Figure 6B; Supplemental Movie 5, right). One interpretation of these data would be that macrophages were physically prevented from interacting with FN when LAM is present. When fluorescently labeled ECMs (Rhodamine-FN and HiLyte 488-LAM) were used in this assay (both at 10 µg/ml) we detected a strong intracellular Rhodamine-FN localization in the presence of LAM (Figure 6C), suggesting that macrophages in this context can interact with FN. Both ECM components were functionalized well on glass when mixed (Supplemental Figure S3C), arguing against the interpretation that the mixed ECM phenotype is due to only LAM being effectively deposited on the surface. These findings support the notion that interactions between specific integrins and distinct elements of the ECM drive motile behavior by differentially regulating cytoskeletal organization and function.

**Figure:** Movie S5 WT macrophages, random migration on 10 μg/mL fibronectin + 10 μg/mL laminin (left), 10 μg/mL fibronectin + 30 μg/mL laminin (middle), 30 μg/mL fibronectin + 10 μg/mL laminin (right).

### Modulation of RGD-based adhesion is sufficient to shift cells on FN to LAM-like phenotype

**FIGURE 7:**
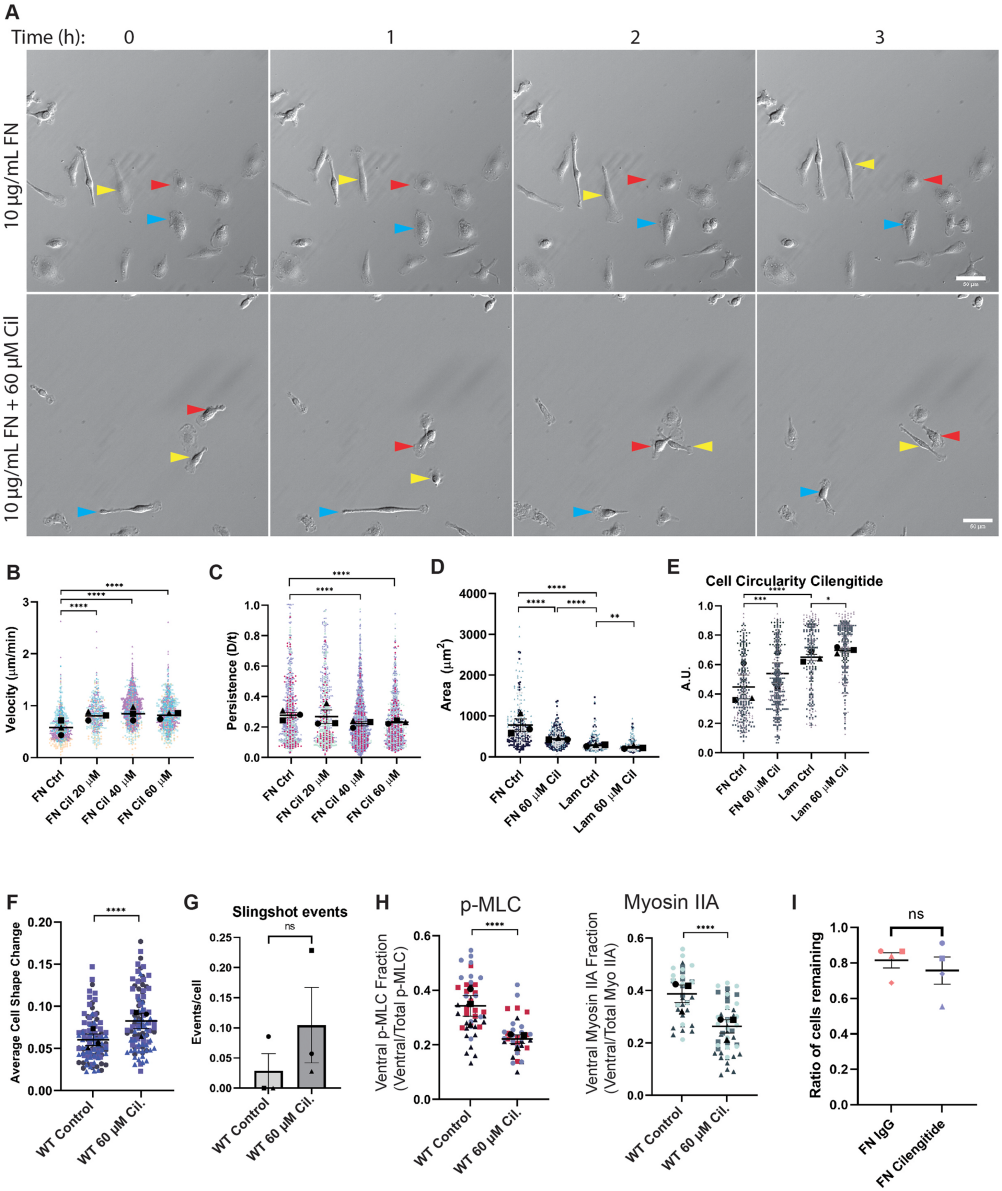
Modulation of RGD-based adhesion is sufficient to shift cells on FN to LAM-like phenotype. (A) Represen­tative relief contrast images demonstrating random migration of FN-plated macrophages treated with DMSO or 60 μM Cilengitide (“Cil”), an RGD-binding integrin inhibitor. Blue, red, and yellow arrowheads in relief contrast images mark different individual cells over the 3-h period. These are part of a longer 16-h time lapse experiment. The unabridged, uncropped data can be found in Supplemental Movie 11. Images in this panel correspond to frames 33, 39, 45, and 51 of the movie. Scale bar = 50 microns. (B) Velocity (cell speed) and (C) persistence (d/T) for macrophages migrating on FN in the presence of the indicated dose of Cilengitide. Means and standard error of the mean for each experiment are represented with black symbols. All data points are plotted and each experimental run is color-coded. Statistical analysis was Kruskal−Wallis with Dunn multiple comparisons test. ****p < 0.0001; FN Ctrl *n* = 716 tracks, FN Cil 20 μM *n* = 463 tracks, FN Cil 40 μM *n* = 1696 tracks, FN Cil 60 μM *n* = 883 tracks, pooled from three experiments. (D) Spread cell area of macrophages on FN or LAM ± 60 μM Cilengitide. Means and standard error of the mean for each experiment are represented with black symbols. All data points are plotted and each experimental run is color-coded. Statistical analysis was Kruskal-Wallis with Dunn multiple comparisons test. ****p < 0.0001, **p = 0.0060; FN Ctrl *n* = 315 cells, FN Cil 60 μM *n* = 359 cells, Lam Ctrl *n* = 255 cells, Lam Cil 60 μM *n* = 277 cells pooled from three experiments. (E) Circularity values for macrophages on FN or LAM ± 60 μM Cilengitide. Means and standard error of the mean for each experiment are represented with black symbols. All data points are plotted and each experimental run is color-coded. Statistical analysis was done by Kruskal–Wallis with Dunn multiple comparisons test. ****p < 0.0001, ***p = 0.0002, *p = 0.0406; FN Ctrl *n* = 315 cells, FN Cil 60 μM *n* = 359 cells, Lam Ctrl *n* = 255 cells, Lam Cil 60 μM *n* = 277 cells, pooled from three experiments. (F) Changes in cellular circularity occurring from frame to frame (10-min intervals) during random motility over the course of 5 h. Means of each experiment and standard error of the mean are represented with black symbols, and all data points are plotted and each experimental run is color-coded. Statistical analysis was Mann−Whitney. ****p < 0.0001; *n* = 106 cells pooled from three experiments. (G) Number of slingshot events per cell in each condition, when analyzed during the 5-h intervals used to generate data in Figure 7F. Events/cell was determined for each experimental run, and are plotted here with the standard error of the mean. (H) Quantification of myosin IIA and p-MLC fluorescence level in the lowest confocal z-slice relative to the total amount of fluorescence staining in the cell. Means for each experiment and standard error of the mean are represented with black symbols, all data points are plotted and each experimental run is color-coded. Statistical analysis was evaluated with an unpaired *t* test for p-MLC data, and Mann−Whitney test for myosin IIA data because that dataset was not normally distributed. ****p < 0.0001 in each case. *n* = 49 cells for p-MLC WT, 36 cells for p-MLC cilengitide, 37 cells for Myosin IIA WT, and 51 cells for Myosin IIA cilengitide conditions, pooled from three experiments. (I) Macrophages plated on 10 μg/ml FN were subjected to gentle washing after treatment with IgG or 60 μM cilengitide. Data is reported as ratio of cells remaining after wash (1 = 100% adherence). An unpaired *t* test was used to determine statistical significance. *P* value = ns, not significant, *N* = 4 independent experiments.

The results from mixed ECM experiments indicate that modulating integrin engagement alone may be sufficient to shift cellular behavior through differential cytoskeletal regulation. We decided to test this idea further by determining whether disruption of RGD-based adhesion to FN rendered cells in that context more “LAM-like”. The small-molecule cilengitide disrupts RGD-binding integrins, most notably αv-containing integrin pairs (Mas-Moruno *et al.*, 2010). Time lapse experiments demonstrated that cilengitide-treated macrophages were small and highly dynamic compared with their untreated counterparts on FN (Figure 7A; Supplemental Movie 6, left). These experiments revealed a significant dose-dependent increase in cell speed and decrease in persistence in cilengitide-treated cells compared with control macrophages on FN (Figure 7. B and C), reminiscent of the LAM motility phenotype. Furthermore, cilengitide-treated macrophages on FN showed a LAM-like decrease in spread cell area and shift toward a more circular morphology (Figure 7, D and E). Frame-to-frame macrophage cell shape changes on FN were increased by cilengitide treatment, though in a much more modest manner than elicited by LAM (Figure 7F), and cilengitide treatment did not cause macrophages to initiate slingshot motility on FN (Figure 7G). In the presence of cilengitide, myosin II and p-MLC were localized throughout the cell (similarly to LAM cells at baseline) rather than contained at the ventral surface (Figure 7H). Despite striking similarities, these data demonstrate that cilengitide treatment on FN does not perfectly phenocopy LAM. The effect of cilengitide cannot be chalked up to altered adhesive strength, as cilengitide does not impair macrophage adhesion to FN (Figure 7I). This suggests that there are other integrins or integrin-independent adhesion systems not targeted by cilengitide that mediate FN adhesion without stimulating spreading. Taken together, our data suggest that it is possible for macrophages to tune their migratory behavior according to which surface receptors interact with the ECM microenvironment.

**Figure:** Movie S6 WT macrophages, random migration on 10 ug/mL laminin + 60 μM cilengitide (left), 10 ug/mL laminin + 40 μM cilengitide + 20 μM Y‐27632 (middle), and 10 ug/mL laminin + 40 μM cilengitide + 15 μM blebbistatin (right).

**FIGURE 8:**
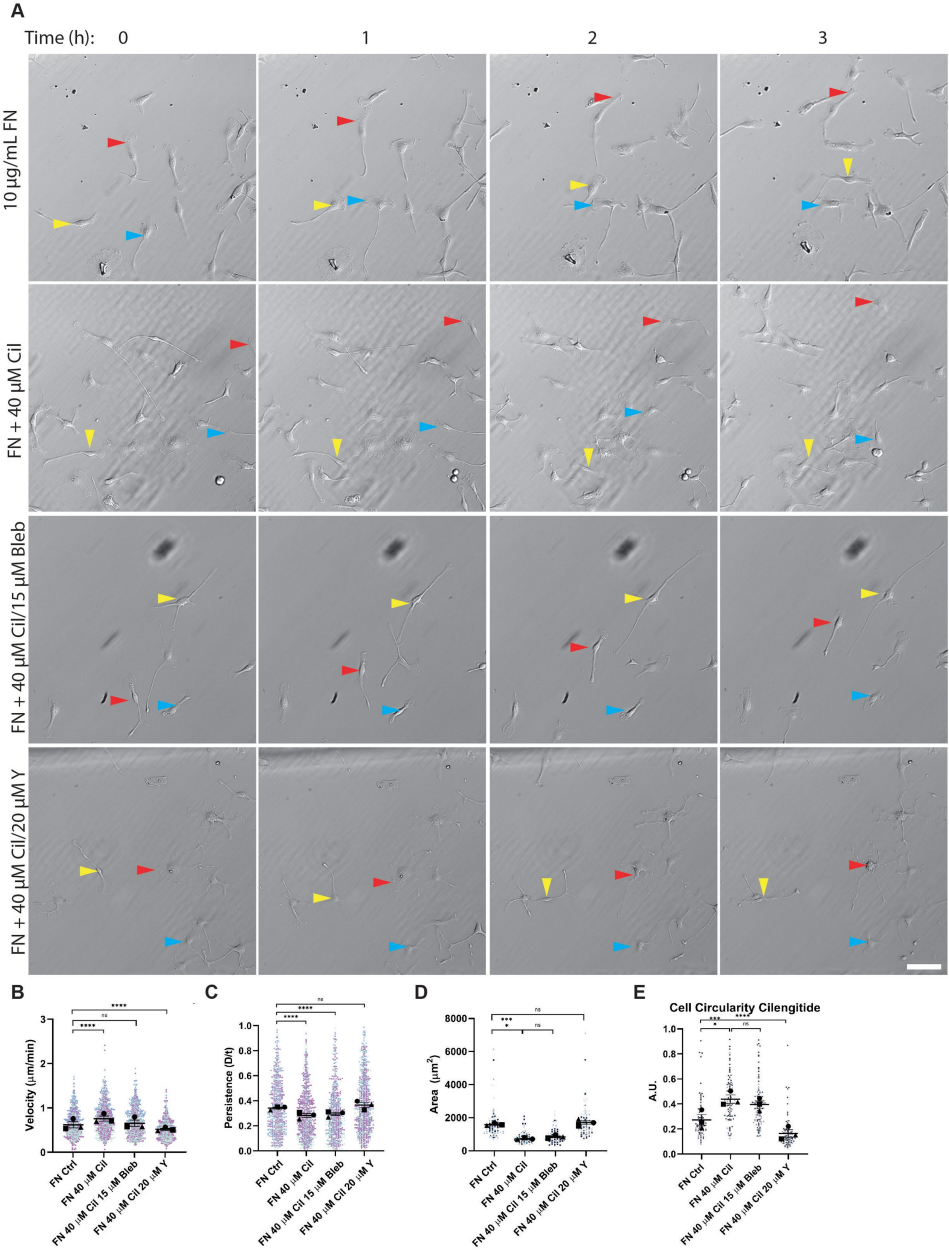
Inhibition of myosin II and ROCK ameliorates cilengitide’s effect on FN motility. (A) Representative relief contrast images demonstrating random migration of FN-plated macrophages treated with DMSO, 40 μM cilengitide (“Cil”), 40 μM cilengitide (“Cil”) + 15 μM Blebbistatin, or 40 μM cilengitide (“Cil”) + 20 μM Y-27632 (“Y”). Blue, red, and yellow arrowheads in relief contrast images mark different individual cells over the 3-h period. These are part of a longer time lapse experiment. The unabridged and uncropped data can be found in Supplemental Movies 12 and 13. Images in this panel correspond to frames 1, 7, 13, and 19 of the movies. Scale bar = 100 µm. (B) Velocity (cell speed) and (C) persistence (d/T) for macrophages migrating on FN in the presence of the indicated inhibitor. Means and standard error of the mean for each experiment are represented with black symbols. All data points are plotted and each experimental run is color-coded. Statistical analysis was done by Kruskal–Wallis with Dunn multiple comparisons test. ****p < 0.0001, FN Ctrl *n* = 735 tracks, FN Cil *n* = 891 tracks, FN Cil + Bleb *n* = 696 tracks, FN Cil + Y *n* = 798 tracks, pooled from three experiments. (D) Spread cell area of macrophages on FN, in presence of indicated inhibitor. Means and standard error of the mean for each experiment are represented with black symbols. All data points are plotted and each experimental run is color-coded. Statistical analysis was done by Kruskal–Wallis with Dunn multiple comparisons test. ****p < 0.0001, FN Ctrl *n* = 105 cells, FN Cil *n* = 105 cells, FN Cil + Bleb *n* = 105 cells, FN Cil + Y *n* = 103 cells, pooled from three experiments. (E) Circularity values for macrophages on FN in presence of indicated inhibitor. Means and standard error of the mean for each experiment are represented with black symbols. All data points are plotted and each experimental run is color-coded. Statistical analysis was done by Kruskal–Wallis with Dunn multiple comparisons test. ****p < 0.0001, FN Ctrl *n* = 105 cells, FN Cil *n* = 105 cells, FN Cil + Bleb *n* = 105 cells, FN Cil + Y *n* = 103 cells, pooled from three experiments.

Myosin II and ROCK are major contributors to the high motility/low persistence LAM phenotype. We next asked whether cilengitide’s ability to increase motility and suppress persistence on FN was also due to these factors. ROCK inhibition corrected the FN cilengitide phenotype most dramatically. Treatment with 20 µM Y compound suppressed motility in the presence of cilengitide and returned persistence values back to WT levels (Figure 8, A–C; Supplemental Movie 6, middle). ROCK inhibition with Y compound also allowed cilengitide-treated cells to spread and become less circular (Figure 8, D and E). Treatment with 15 µM blebbistatin, a dose that does not impact macrophage motility on FN at baseline (Supplemental Figure S3A), also impacted the cilengitide phenotype. As we anticipated, treatment with blebbistatin in the presence of cilengitide brought macrophage cell speed down to control FN levels, but had little effect on persistence (Figure 8, A–C; Supplemental Movie 6, right). In addition, blebbistatin did not spread or elongate cilengitide-treated macrophages on FN (Figure 8, D and E). These outcomes are similar to the effect of blebbistatin on LAM migration. We conclude, based on these results, that impairing RGD-integrin interactions causes macrophages on FN to become more LAM-like, adopting morphology and motility phenotypes that require myosin II and ROCK activity.

## DISCUSSION

Macrophages express a variety of integrins that bind different ECM components in vivo, though the influence of each ECM component on macrophage signaling is not fully known. The large array of integrins expressed by macrophages may give rise to differential signaling based on how a macrophage “reads” the ECM in its microenvironment. This study offers the distinction between how macrophages respond to FN and LAM as proof of principle that macrophages can discriminate between distinct ECM components. While the observation that macrophages are faster on LAM than FN has been made before (Wheeler *et al.*, 2006), little is known about what governs the striking motility shift. We herein provide a molecular explanation for this behavior. RGD-based adhesion to FN induces macrophages to flatten and spread, as well as biasing myosin IIA to the adhesive plain of the cell. Such an arrangement corresponds to well-organized cellular protrusions, yielding relatively persistent but slow whole cell migration. On the other hand, adhesion to LAM elicits an inverse effect wherein macrophages fail to spread and become rounder, with myosin IIA more evenly dispersed throughout the cell. Myosin IIA organized in this way is what drives macrophage slingshot motility on LAM, leading to less persistent, but faster, migration. This shift is remarkably similar to the myosin-dependent slingshot motility demonstrated by mesenchymal cells migrating in deformable three-dimensional matrices (Wang *et al.*, 2019). Inhibition of myosin IIA and ROCK together render LAM-plated cells more “FN-like”. It is also possible to shift macrophages on FN to a more “LAM-like” behavior simply by impairing RGD-binding integrins, which again correlates with a redistribution of myosin IIA throughout the cell similar to its distribution on LAM. We have summarized our major findings in graphical form in Supplemental Figure S3D.

These findings suggest a fundamental link between ECM-dependent activation of specific integrins and the actomyosin cytoskeleton. The FN-binding α4 integrin is known to interact with myosin IIA (Rivera Rosado *et al.*, 2011). FN also stimulates an integrin- and Rac1-dependent localization of myosin IIA to focal adhesions (Pasapera *et al.*, 2015), suggesting a link between cell spreading (via Rac1-Arp2/3 complex) and actomyosin localization reminiscent of our macrophages on FN. This localization is mechanically important for motile cells, as myosin II plays a major role in focal adhesion formation and traction generation on FN (Schiller *et al.*, 2013). These findings suggest that perturbing the integrin-FN interaction, as with cilengitide, should alter the localization of myosin IIA in line with findings in the present study. Our data also implicates ROCK in the LAM phenotype. Previous work has noted that ROCK activity increases the height of the actin cortex in mesenchymal cells (Grandy *et al.*, 2022), which is thought to be due to enhanced cytoskeletal tension. The ROCK-dependent redistribution of myosin IIA away from the bottom of the cell in the presence of cilengitide or when plated on LAM could lead to higher cortical contractility, which could drive the fast, meandering motility style seen in this context.

Macrophages spread poorly on LAM and migrate faster, but less persistently on it, despite expressing the α6β1 integrin pair (as seen in previous studies [Shaw *et al.*, 1990; Wei *et al.*, 1998; Guo *et al.*, 2015]). This integrin pair is active to some extent on LAM, as blocking α6 function led to even more severe adhesion defects on LAM. Activation of this integrin pair (or its regulation in this context) may have altogether different effects compared with FN-binding integrins. As macrophage behavior on LAM is a durable and regulated response, it seems likely that specific cellular behaviors are stimulated (or impaired) by α6β1-LAM ligation. Along these lines, a previous study using neuronal cells suggested that LAM might impair focal adhesion formation (Calof and Lander, 1991). The current study supports and expands upon this notion by demonstrating that macrophages preferentially respond to LAM when both LAM and FN are made available for adhesion. This response is surprisingly durable, as we have not been able to demonstrate a concentration of FN that definitively “wins” over LAM. We propose a model wherein LAM retains its “dominance” in this context via inhibitory crosstalk with RGD-binding integrins. Plentiful evidence exists for integrin crosstalk in a variety of contexts (Porter and Hogg, 1997; Riederer *et al.*, 2002; Orr *et al.*, 2006; Longmate *et al.*, 2017). α6β1 could enforce inhibitory crosstalk at the level of specific kinases that suppress RGD-binding integrins, factors that weaken nascent FN-responsive adhesions by directly organizing myosin II into the cortex rather than adhesions, or by outcompeting RGD integrins for talin or other focal adhesion components. Previous literature suggests the compelling possibility that protein kinase C (PKC) regulation may determine ECM-specific macrophage behavior. An extensive body of literature links PKC to adhesion, especially on FN (Jaken *et al.*, 1989; Woods and Couchman, 1992; Vuori and Ruoslahti, 1993; Liliental and Chang, 1998; Ng *et al.*, 1999). However, PKC activity is not inherently stimulated in macrophages on LAM, requiring exogenous activation by PMA or cytokine to induce strong adhesion (Shaw and Mercurio, 1989; Shaw *et al.*, 1990). Curiously, this PMA-induced adhesion is still dependent upon α6β1 integrin (Shaw *et al.*, 1990). These studies suggest that there may be a LAM-responsive negative regulator of PKC activation that alters inside-out signaling and actively prevents strong adhesion unless an overriding stimulus is present. PKC inhibition by a LAM-responsive factor could also explain LAM’s dominance over FN in this study. As a whole, this work argues that the LAM phenotype arises from an active process initiated and maintained by α6β1-LAM ligation rather than macrophages simply being less adherent to LAM. These observations are worthy of additional investigation.

Each local tissue microenvironment houses competing cues that together influence the wide range of macrophage behaviors, especially in the context of trauma and infection (Avraham *et al.*, 2015; Yunna *et al.*, 2020). Furthermore, ECM composition can be highly variable in a spatial or temporal sense. For example, different areas of the same tissue may have distinct ECM contexts. The basement membrane of epithelial cells contains high concentrations of LAM, whereas the interstitial connective tissue underneath contains FN and Col. It may be possible to track cellular responses in vivo or ex vivo and correlate them with proximity to regions known to have a particular ECM context. These studies could reveal that there are subpopulations of macrophages that look similar from a transcriptional perspective, but respond very differently to external stimuli due to being surrounded by different ECM contexts. It seems fitting that the inherently adaptable macrophage would be capable of immediately responding to new ECM environments, presumably without requiring transcriptional changes. It remains to be seen how ECM sensing alters other elements of macrophage function beyond motility, and whether in vivo evidence of cells undergoing ECM-sensitive behavioral shifts can be found.

There is clear precedence for ECM-dependent immunomodulation, though there is likely much mechanistic detail yet to be uncovered. For example, FN is heavily associated with wound healing and repair (Grinnell, 1984; Logie *et al.*, 2021). However, the literature suggests that some FN isoforms are associated with inflammation while others have an anti-inflammatory influence (Grinnell, 1984; Ffrench-Constant *et al.*, 1989; Jarnagin *et al.*, 1994). Fibrinogen was recently found to prime macrophages toward a pro-inflammatory phenotype, whereas the cleavage of fibrinogen to form fibrin resulted in anti-inflammatory polarization (Hsieh *et al.*, 2017). As the cytoskeleton is intimately involved in sensing ECM, it is likely that these immunomodulatory shifts can be linked to differential cytoskeletal regulation by specific ECM microenvironments. ECM contexts also change during pathological conditions, with tumorigenesis being one notable example. Tumor-associated macrophages, which must respond to an evolving ECM microenvironment, may offer a relevant in vivo setting where the ability to distinguish and respond to distinct ECM components has crucial implications for disease progression and prognosis.

## MATERIALS AND METHODS

Request a protocol through *Bio-protocol*.

### BMDM

Hematopoietic cells were isolated from mouse femurs. Bone marrow was flushed from the bone, filtered to prepare single cell suspensions, and then cultured in “macrophage media” composed of 70% DMEM complete (containing a final concentration of 10% fetal bovine serum [FBS], 1% penicillin/streptomycin, and 1% Glutamax) + 30% L929-conditioned media (for a source of macrophage colony stimulating factor [M-CSF]) for at least 7 d before experiments at 37°C, 90% humidity, and 5% CO_2_. Differentiated bone marrow macrophages were sorted from this initial bulk population on the basis of F4/80 positivity using an Alexa 488–conjugated antibody and FACS sorting. For all experiments contained in the present study, macrophages were maintained in macrophage media at 37°C, 90% humidity, and 5% CO_2_. Macrophages were passaged at 60–90% confluency. They were washed once with 1X phosphate-buffered saline (PBS), then incubated at 4°C for 10 min with prechilled 0.5 mM ethylenediaminetetraacetic acid (EDTA). At the end of the incubation EDTA was removed and macrophages were scraped directly into 1–3 ml macrophage media (according to desired confluency after passage) and replated for passaging and/or experimentation. For specific experiments, as noted, human and mouse macrophages were cultured in defined, serum-free macrophage media (Thermo Fisher Scientific, catalogue# 12065074).

### Mouse lines used to derive macrophages

The BMDM used in this study come from a mixed strain (though predominantly C57BL/6) of mice harboring a conditional *Arpc2* allele (Rotty *et al.*, 2017). This allele consists of LoxP sites flanking exon 8 of the gene encoding the p34/Arpc2 subunit of the Arp2/3 complex. In addition, these cells harbor *Ink4a−/−*; *Arf−/−* alleles, which allow them to persist in culture without becoming senescent. This is a defined genetic event rather than a spontaneously arising oncogenic immortalization. Finally, these cells also harbor a Rosa26-CreER transgenic allele that allows for conditional deletion of *Arpc2* exon 8 in the presence of tamoxifen. None of the cells used in this study were exposed to tamoxifen, so they were considered functionally wild-type for the duration of their time in culture. This combination of alleles has previously been published (Rotty *et al.*, 2017; Pipathsouk *et al.*, 2021; Ronzier *et al.*, 2022).

### Human primary monocyte differentiation and culture

Primary human monocytes were donated to our lab by Luxia Zhang. Human monocytes were incubated at a concentration of ∼5 × 10^5^ cells/ml in complete RPMI with L-glutamate (Thermo Fisher Scientific, catalogue #11875093; plus 10% heat-inactivated FBS and 1% penicillin/streptomycin, Sigma #P4333-100ML) and 50 ng/ml recombinant human M-CSF (STEMCELL Technologies, #78057.1) for at least 7 d at 37°C, 90% humidity, and 5% CO_2_. Media was changed every 3–4 d. Cells were passaged as necessary during differentiation and subsequent experiments by incubation with 0.05% Trypsin-EDTA (Caisson Labs, #TRL02-100ML) for 10 min at 37°C, followed by resuspension in complete RPMI with M-CSF.

### Reagents

Antibodies: α6 integrin antibody (GoH3, Thermo Fisher Scientific, #14-0495-82); p-Cofilin Ser3 antibody (77G2, Cell Signaling Technology, #3313); p-MLC Ser20 (for Western blot analysis; Thermo Fisher Scientific, #MA5-27983); GAPDH antibody (clone 6C5, Thermo Fisher Scientific, #AM4300); Myosin IIA antibody (Polyclonal, Cell Signaling Technology, #3403); p-MLC 2 antibody (for immunofluorescence; Polyclonal, Cell Signaling Technology, #3674); FN (Abcam, ab2413); Goat antirabbit IgG Rhodamine Red-X (RRX) secondary antibody (Polyclonal, Jackson Immunoresearch, 111-295-144); Goat antimouse IgG RRX secondary antibody (Polyclonal, Jackson Immunoresearch, 115-295-166); Goat antirabbit IgG Alexa 488 secondary antibody (Polyclonal, Jackson Immunoresearch, 111-545-144); Goat antimouse IgG Alexa 488 secondary antibody (Polyclonal, Jackson Immunoresearch, 115-545-166); Goat antirat IgG RRX secondary antibody (Polyclonal, Jackson Immunoresearch, 112-295-167); Goat antirat IgG Alexa 488 secondary antibody (Polyclonal, Jackson Immunoresearch, 112-545-003); Goat antimouse IgG, HRP-conjugated (Jackson Immunoresearch, 115-035-146); Goat antirabbit IgG, HRP-conjugated (Jackson Immunoresearch, 111-035-144)

ECMcomponents: (P-L-L; Sigma-Aldrich, #P8920); Rat tail Col, type I (Thermo Fisher Scientific, #A1048301); FN, human plasma (Thermo Fisher Scientific, #33016015); LAM 111, mouse (Thermo Fisher Scientific, #23017015); VN, human plasma (Sigma-Aldrich, #5051); Rhodamine-conjugated FN (Cytoskeleton, FNR01-A); HiLyte 488-labeled LAM (Cytoskeleton Inc., LMN02-A)

Other reagents: CellTracker Green CMFDA Dye (Invitrogen, #C7025); CellBrite Orange: Ex/Em 549/565 nm (Biotium, #30022); Alexa Fluor 647 Phalloidin (Invitrogen, #A22287); Alexa Fluor 488 Phalloidin (Invitrogen, #A12379)

### Inhibitors and Inhibitor Use

S-nitro blebbistatin (at 7.5, 15, and 30 µM; Thermo Fisher Scientific NC0664123), Y-27632 (at 5, 10, and 20 µM; Abcam ab120129), cilengitide (at 20, 40, and 60 µM; Sigma-Aldrich SML 1594-5MG). Inhibitor dilutions were made up in macrophage media. Cells were exposed to inhibitor for at least 1 h before initiation of experiment, and inhibitor was present throughout the duration of the experiment unless otherwise noted.

### Immunofluorescence

Twelve-millimeter glass coverslips were coated inside single wells of 24-well dishes, and coated with 400 µl of ECM diluted to desired concentration in sterile 1X PBS. Coverslips were incubated for 1 h at 37°C. After 1 h, coverslips were washed three times with sterile 1X PBS. After final wash, 400 µl of macrophage media was added to each well. Each coated coverslip was seeded with 7500–10,000 macrophages, depending on the experiment. Macrophages were allowed to equilibrate and spread overnight in the incubator. Media was gently aspirated off the following day, leaving cells bound to the coverslip. Cells were washed once with room temperature (RT) nonsterile 1x PBS. Cold or RT (depending on antigen) 4% paraformaldehyde in Krebs Buffer or PBS (depending on antigen) was used to fix cells; incubation was 10 min at room temperature. Wells were then washed 3 × 10 min with 2 ml PBS. PBS was aspirated and cells were permeabilized for 5 min with 0.1% Triton X-100 in PBS. Coverslips were again washed rapidly three times with PBS. Coverslips were then incubated for 30 min at room temperature with a blocking solution of 5% BSA and 5% normal goat serum in PBS and then aspirated. Primary antibody was diluted into 1% BSA in PBS (range of 1:100–1:250, depending on antibody). Coverslips were then incubated for 1 h at room temperature. Coverslips were then washed 2 × 5 min in RT PBS. Fluorescent secondary antibodies and phalloidin were diluted 1:500 into 1% BSA in PBS and incubated for 30 min at room temperature. Secondary antibody solution was then aspirated and wells were washed with 1:10,000 Hoechst diluted in PBS for 5 min at RT. The wells were washed once more with PBS for 5 min at RT. After the final 1x PBS wash, 2 µl of fluoromount G was added to a microscope slide. Using fine forceps, each coverslip was transferred cell-side-down to the fluoromount G. After a 10-min incubation at room temperature in the dark, the coverslips were sealed with nail polish. Samples were then imaged on an Olympus IX83 epifluorescence microscope, a Zeiss 700 confocal microscope, or a Zeiss 980 Airyscan (see details, below).

### Time-lapse video microscopy

Col, FN, LAM, VN, and P-L-L were diluted to the desired concentration in sterile 1X PBS. Glass bottomed eight-well chamber slides were coated with 300 µl of the appropriate working dilution of ECM. Chamber slides were then incubated at 37°C for 1 h. ECM solution was then aspirated and surfaces washed rapidly three times with sterile 1X PBS. Macrophage media was added to each chamber, and the chamber was stored in the incubator until cells were ready to be added. Labeling with tracker dye: A 1:10,000 dilution of green cell tracker dye in PBS, or 1:1000 dilution of CellBrite Cytoplasmic Membrane Dye Orange (Biotium #30022) was made in macrophage media. Macrophage media was aspirated from the culture dish and working solution of green cell tracker dye was added at room temperature for 5 min, and then handled according to standard macrophage passaging protocol. If orange cell tracker dye was used, the working solution was added to culture dish for 1 h, and then cells were handled according to standard macrophage passaging protocol. Seeding macrophages in chambers: After labeling, 5000 cells (mouse) or 10,000 cells (human) were added to each well of the culture chamber. Mouse primary macrophages were immediately moved to IX83 Olympus microscope and loaded a Tokai Hit stage-top environmental chamber set to keep the cells at 5% CO_2_ and 37°C (see details below). Human primary macrophages were returned into the incubator overnight, and moved to the IX83 Olympus microscope the next day. Cells were allowed to equilibrate for at least an hour before imaging. Cells were then imaged at 20x magnification via relief contrast and the appropriate channel for the chosen tracker dye. In a typical experiment seven positions in each condition were chosen. The time lapse was set to image every 10 min for at least 16 h. Image analysis to obtain migration velocity and persistence was performed in FIJI. The TrackMate plugin was used to generate track information, which was subsequently loaded into the Chemotaxis plugin to generate velocity and persistence values. The only exception to this is cells treated with blebbistatin alone, which were not fluorescently labeled due to blebbistatin’s cytotoxicity under blue light illumination as these experiments occurred before our adoption of orange tracker dye. These experiments were tracked by hand using the FIJI manual tracking plugin rather than TrackMate, but subsequent analysis with the Chemotaxis tool occurred identically to the procedure outlined above. More detail can be found in the quantitative image analysis section, below.

### Adhesion assays

Glass surfaces of multiwell chamber slides were coated with 10 µg/ml FN or 10 µg/ml LAM for 1 h at 37°C. Chambers were then washed three times with sterile 1X PBS and stored overnight at 4°C. PBS was aspirated the next morning at 300 µl macrophage media was added, and chamber was allowed to equilibrate in a tissue culture incubator for at least 1 h before addition of cells. Inhibition with α6 blocking antibody: 20,000 macrophages were centrifuged for 4 min at 1000 × *g*. Excess media was aspirated and cells were resuspended in 15 µl of 2% FBS containing a 1:1000 dilution of orange cell tracker dye, and either α6 blocking antibody or isotype normal IgG control. Macrophages were resuspended and incubated for 37°C for 60 min, and were vortexed once halfway through incubation. Macrophages were centrifuged again for 4 min at 1000x g and resuspended in 10 µl of macrophage media. Five microliter of this suspension (∼10,000 cells) was added to previously coated chamber wells. Cilengitide treatment: Cells were treated according to standard passaging protocol. Ten thousand cells were plated directly into chamber wells containing Cilengitide or an equal volume of DMSO. Imaging and analysis: After either approach, the chamber slide was transferred to the BioTEK Cytation microscope. In each case cells were allowed to adhere and spread for 2 h. Images were collected at this point, representing the “prewash” population. Macrophage media was then aspirated and two washes with 1X PBS were used to gently wash cells on the cover glass with a P1000 pipette. The final wash was aspirated and 300 µl of macrophage media was added back, and another round of imaging was conducted to capture postwash macrophages. Cell number was quantified from the TRITC channel (to detect cell tracker label) before and after washes to obtain the ratio of macrophages before and after washing. Human cells were processed and imaged similarly, except that they were analyzed for adhesion at the end of motility experiments (see above) and were on the coated surfaces for ∼48 h before being subjected to adhesion assay.

### FN Deposition from Serum-Containing Media Quantification

Glass bottomed eight-well chamber slides were coated with either 1X PBS, 10 µg/ml FN, 10 µg/ml LAM, 1.0 µg/ml LAM, or 0.1 µg/ml LAM with or without subsequent 5% BSA backfill, as described above. After chambers were washed, 300 µl of either serum-free macrophage media (in the case of 1X PBS) or standard macrophage media (in all other conditions) was added to each chamber. The slide was incubated at 37°C overnight to mimic environmental conditions for migration assays. The following day, the media was aspirated and the wells were labeled with primary FN antibodies and fluorescent secondary antibodies, as described above. The chambers were filled with PBS and wrapped with parafilm to prevent evaporation. Identical imaging conditions were used for each experimental replicate. Chambers were imaged at 20x magnification with an exposure time of 200 ms. Fluorescence intensity was quantified with ImageJ.

### Quantitative Image Analysis

Live cell velocity and persistence tracking: Image files were opened with the FIJI Bio-Formats Importer plugin. An FFT bandpass filter was used to enhance contrast between cells and the background, and applied with default settings. The FIJI TrackMate plugin was used to identify and track fluorescently labeled cells. Spots are set to color by contrast automatically. LoG detector was selected, and estimated “spot” diameter was set such that as many artifacts as possible were excluded and as many cells as possible were detected. Simple LAP tracker was selected. Linking max distance and gap-closing distance were both set to 70.0. Gap-closing max frame gap was set to 10. Tracks were filtered based on track duration, track mean speed, and track displacement to filter out dead cells and artifacts (which typically had a speed and displacement of 0) and to avoid sampling tracks with overly short durations. Track duration was set to a minimum of 20 frames in order to be included in analysis. Track mean speed was set to at least 2.00 and track displacement was set to at least 8.00. Raw data were saved as .csv files and imported into the Ibidi Chemotaxis tool FIJI plugin to obtain velocity and persistence values for each cell track. Cell area, elongation, and F-actin fluorescence: The Olympus CellSens software was used to quantify cell area, elongation and fluorescence intensity from Phalloidin staining (which labels F-actin throughout the cell). Parameters were set such that small fragments of cells were excluded from batch analysis, and signals arising from multiple cells (e.g., cells touching in an image) were manually excluded from analysis. Circularity quantification: The freehand selection tool in FIJI was used to trace cells in relief contrast images. Circularity ranges from a value of 1 (perfectly circular) to 0 and was determined using the “shape descriptors” function. Internalization of Rhodamine-FN: Images were imported into FIJI with autoscale turned off. Display range is manually set to 0 minimum and 10,000 maximum. These values were kept consistent for images with and without Rhodamine-FN. “Set measurements” was used to measure area, mean gray value, and min and max gray value. Cells were traced in the relief contrast channel, then the channel selector was toggled to the fluorescent channel, where the measurements were taken. Fluorescent ECM coating: Images were imported into FIJI with autoscale turned off. Display range was manually set to 0 minimum and 10,000 maximum value. “Set measurements” was used to determine mean gray value, and min and max gray value. A square size was manually set on the image in the top left corner of each image, then measurement was taken. The square was then moved to the top right corner, the center, bottom left, and bottom right corner, with measurement taken at every position. This was repeated for every concentration condition. Adhesion assay: Macrophages labeled with orange cell tracker dye were automatically detected by the BioTEK Cytation software. Cell counts were taken immediately before and after adherent cells were washed with PBS. Measurements were taken in approximately the same area. The reported value is a simple ratio between the cell count at the end of the assay divided by cell count at the start. Cell shape change quantification: A 5-h window from each overnight time lapse experiment was used for the analysis. The same timeframes were used from each position within an experimental set. The 5-h time lapse session was imported into FIJI. Fluorescent images of tracker dye-labeled cells were processed so that brightness and contrast were high, and fluorescent background was minimized. This allowed the entirety of the cell to be fluorescent label-positive, so that the FIJI magic wand tool could be employed to mark each cell at every time point in a semiautomated manner. Circularity measurements for each cell at each time point were gathered in FIJI. At this point the absolute value of t_n_-t_n+1_ was taken, where *n* = frame number. The average shape change for each analyzed cell was then calculated. The mean of the entire cell population average was then quantified. Blebbistatin-treated cells were not fluorescently labeled, due to blebbistatin’s known cytotoxicity under blue light illumination. The analysis process for these samples was largely the same, except that the relief contrast channel was used for analysis and cell boundaries were outlined by hand. All subsequent analysis was done identically to the procedure outlined above. Slingshot behavior quantification: A single-frame cell shape change value of 0.4 or higher during the 5 h analysis window was defined as a “slingshot event”. This is based on empirical observation of slingshot events in comparison to known cell shape change values calculated during the event. Every cell shape change value for every cell in a population (e.g., WT cells on FN) was sorted into one dataset. The number of values higher than 0.4 was recorded and divided by the number of cells in the population. This single value was reported for each population in each experiment conducted. Myosin II organization: Macrophages on FN or LAM were fixed with 4% paraformaldehyde and processed for microscopy as indicated above. Myosin IIA and p-MLC antibodies were used for analysis in this experiment. Individual confocal slices were hand outlined in FIJI to capture all myosin II or p-MLC signal from each slice of the z-series, and integrated pixel density was quantified. “Ventral” myosin II was defined as the pool of myosin in the cell’s lowest z-frame (i.e., the side of the cell closest to the coverslip). Myosin II or p-MLC localizing to the bottom of the cell was expressed as a fraction of the total intensity across all z-slices of the cell. A larger value indicates that more of the myosin II or p-MLC signal localizes to the cell bottom.

### Microscopes

Epifluorescence: Epifluorescence of fixed images was done at room temperature on an Olympus IX83 outfitted with an X-cite 120 LED Boost light source (Excelitas Tech.) for fluorescent imaging. A Hammamatsu digital camera (C13440-20CU) was used to capture images. 10x, 20x, or 100x objectives were used during epifluorescence experiments, depending on experimental needs. Time-lapse video microscopy: Time-lapse video microscopy was conducted on either the Olympus IX83 or BioTEK Cytation, as noted previously in the methods. For the IX83, an INU incubation system controller from Tokai Hit was used to maintain cells in a stable environment at 37°C, 90% humidity, and 5% CO_2_. A 20X objective was employed during time lapse imaging. A BioTEK Cytation 5 was used primarily for adhesion assays. This setup includes a wide field of view camera, high performance imaging controller and a laser autofocus add-on. Imaging can be done at 4X, 10X, or 20X magnification. Confocal: A Zeiss LSM 700 confocal microscope housed at the USUHS Biological Instrumentation Center (BIC) was employed for higher resolution imaging of fixed samples using a 40X oil immersion objective. A Zeiss 980 Airyscan housed at the USUHS BIC was employed for GFP-myosin IIA imaging using a 63X oil immersion objective. All confocal and Airyscan imaging was done with fluorescent filter sets appropriate for each fluorophore.

### Western blotting

Protein lysis: Macrophages were washed three times with ice-cold PBS. After final PBS aspiration, cells were kept on ice and RIPA buffer (Sigma R0278-50ML) with 1X protease inhibitor (Thermo Fisher Scientific A32953) and 1X phosphatase inhibitor (Sigma 4906845001) was added to cells. Cells were then scraped thoroughly and transferred to Eppendorf tubes, which were kept on ice for 15 min. Tubes were then centrifuged at 4°C in a prechilled microcentrifuge for 10 min. The supernatant was transferred to a new tube and the pellet was discarded. Precision Red (Cytoskeleton ADV02-A) was used to quantify protein amount in RIPA lysate. Analysis was done via plate reader. Samples were prepared based on protein reading, and typically 8 µg of protein sample was loaded per well for electrophoresis. Electrophoresis: Samples were combined with 1X Bolt LDS Sample Buffer (Thermo Fisher Scientific B0007) and 1X NuPAGE Sample Reducing Agent (Novex NP0009), and remaining volume was made up with excess RIPA buffer to ensure that both an equal volume and protein concentration was maintained across samples. Samples were vortexed briefly, heated to 70°C for 10 min, and centrifuged at max speed for 1 min. Samples were then loaded onto a Bolt 4–12% Protein Gel (Thermo Fisher Scientific NW04125BOX). The gel electrophoresis chamber was filled with 1X NuPAGE MES SDS Running Buffer (Thermo Fisher Scientific NP0002) and run at 200 V. Electrophoresis was stopped when the loading dye front reached the bottom of the gel. Transfer: A traditional wet transfer to PVDF membrane (Bio-Rad 1620174) was done for 1 h on ice with chilled transfer buffer using 0.35 A (constant amps setting). Transfer buffer was diluted to 1X from 10X stock (for 1L of 10X transfer buffer: 19.70 g Trizma HCl, 15.14 g Trizma base, 142.63 g glycine, adjusted to pH 8.3). After dilution methanol was added to a final concentration of 20%. Blotting: Membranes were placed directly in 5% milk (in 1X Tris-buffered saline + Tween [TBST]) for 1 h at room temperature. Membranes were then washed, and primary antibody diluted in 5% BSA (in 1X TBST) or 5% milk (in 1X TBST) was added overnight in the cold room with gentle agitation. Membranes were then washed 3 × 5 min with 1X TBST. The membrane was then incubated for 30 min with 5% milk in TBST containing a 1:10,000 dilution of HRP-conjugated secondary antibody. The blot was then washed 3 × 5 min with 1X TBST. HRP signal was activated with SuperSignal West Pico Chemiluminescent Substrate and imaged on an Amersham Imager 680. FIJI was used to analyze band intensity, using identically sized boxes to determine total pixel intensity in each sample for each protein of interest. All intensities were normalized to GAPDH to control for differences in loading across samples. Uncropped blots can be found in Supplemental Figure S5. Images were evaluated to ensure that only unsaturated exposures were analyzed. Any brightness and contrast changes were applied equally to the entire image.

### Statistical analysis

The Kruskal–Wallis with Dunn multiple comparisons test was used to assess significance in experiments where a normal distribution of the dataset could not be assumed. When only two experimental conditions were tested, we used Mann–Whitney test when we could not assume normality. Unpaired *t* tests and ANOVAs were used when normality tests indicated a normal distribution of the data. All statistics were calculated using GraphPad Prism, and significance was assumed if *p* ≤ 0.05. More information on each statistical test can be found in the relevant figure legend panel.

### Data availability statement

All primary data will be openly available upon request.

## Supplementary Material



## Abbreviations used:

Arp: actin related protein
BMDM: bone marrow-derived macrophage
Cil: cilengitide
Col: collagen
ECM: extracellular matrix
FN: fibronectin
LAM: laminin 111
M-CSF: macrophage colony-stimulating factor
MLC: myosin light chain
Myo IIA: myosin IIA
P-L-L or PLL: poly- l-lysine
RC: relief contrast
Rhod: rhodamine
RRX: Rhodamine Red-X
RT: room temperature
SFM: serum-free media
VN: vitronectin
Y or Y compound: Y-27632
