# Supplementary material for: Macrophage migration is differentially regulated by fibronectin and laminin through altered adhesion and myosin II localization
Source: Mol Biol Cell. 2024 Jan 12;35(2):ar22. doi: 10.1091/mbc.E23-04-0137 (PMC10881148; doi:10.1091/mbc.E23-04-0137)
Supplement: Supplementary file 7 [file mbc-35-ar22-s001.pdf]

Supplemental Materials

Molecular Biology of the Cell

Stinson *et al*.

A

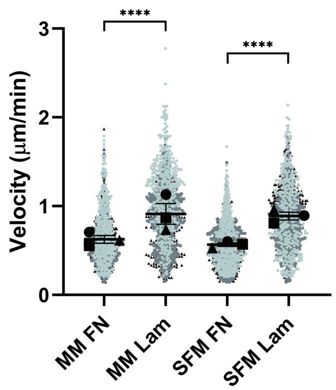

B

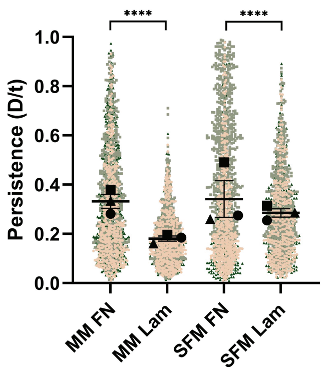

C

Cell Area (Figure 1B)	Summary	p-value
Poly-L-Lysine vs. Collagen	*	0.0163
Poly-L-Lysine vs. Fibronectin	****	<0.0001
Poly-L-Lysine vs. Laminin	****	<0.0001
Poly-L-Lysine vs. Vitronectin	ns	0.695
Collagen vs. Fibronectin	****	<0.0001
Collagen vs. Laminin	****	<0.0001
Collagen vs. Vitronectin	ns	>0.9999
Fibronectin vs. Laminin	****	<0.0001
Fibronectin vs. Vitronectin	****	<0.0001
Laminin vs. Vitronectin	****	<0.0001
Test: Kruskal-Wallis, with Dunn's multiple comparisons		

Elongation (Figure 1C)	Summary	p-value
Poly-L-Lysine vs. Collagen	***	0.0001
Poly-L-Lysine vs. Fibronectin	****	<0.0001
Poly-L-Lysine vs. Laminin	****	<0.0001
Poly-L-Lysine vs. Vitronectin	****	<0.0001
Collagen vs. Fibronectin	ns	>0.9999
Collagen vs. Laminin	****	<0.0001
Collagen vs. Vitronectin	ns	>0.9999
Fibronectin vs. Laminin	****	<0.0001
Fibronectin vs. Vitronectin	ns	>0.9999
Laminin vs. Vitronectin	****	<0.0001
Test: Kruskal-Wallis, with Dunn's multiple comparisons		

F-actin staining (Figure 1D)	Summary	p-value
Poly-L-Lysine vs. Collagen	ns	>0.9999
Poly-L-Lysine vs. Fibronectin	ns	>0.9999
Poly-L-Lysine vs. Laminin	****	<0.0001
Poly-L-Lysine vs. Vitronectin	ns	0.0558
Collagen vs. Fibronectin	ns	>0.9999
Collagen vs. Laminin	****	<0.0001
Collagen vs. Vitronectin	ns	>0.9999
Fibronectin vs. Laminin	****	<0.0001
Fibronectin vs. Vitronectin	ns	0.0524
Laminin vs. Vitronectin	****	<0.0001
Test: Kruskal-Wallis, with Dunn's multiple comparisons		

Cell speed (velocity) (Figure 1F)	Summary	p-value
Poly-L-Lysine vs. Collagen	ns	>0.9999
Poly-L-Lysine vs. Fibronectin	***	0.0004
Poly-L-Lysine vs. Laminin	****	<0.0001
Poly-L-Lysine vs. Vitronectin	ns	0.319
Collagen vs. Fibronectin	****	<0.0001
Collagen vs. Laminin	****	<0.0001
Collagen vs. Vitronectin	*	0.0202
Fibronectin vs. Laminin	****	<0.0001
Fibronectin vs. Vitronectin	ns	>0.9999
Laminin vs. Vitronectin	****	<0.0001
Test: Kruskal-Wallis, with Dunn's multiple comparisons		

Persistence (Figure 1G)	Summary	p-value
Poly-L-Lysine vs. Collagen	ns	>0.9999
Poly-L-Lysine vs. Fibronectin	***	0.0008
Poly-L-Lysine vs. Laminin	ns	>0.9999
Poly-L-Lysine vs. Vitronectin	****	<0.0001
Collagen vs. Fibronectin	**	0.0032
Collagen vs. Laminin	ns	>0.9999
Collagen vs. Vitronectin	****	<0.0001
Fibronectin vs. Laminin	***	0.0001
Fibronectin vs. Vitronectin	****	<0.0001
Laminin vs. Vitronectin	****	<0.0001
Test: Kruskal-Wallis, with Dunn's multiple comparisons		

Stinson et al., Supplemental Figure 1

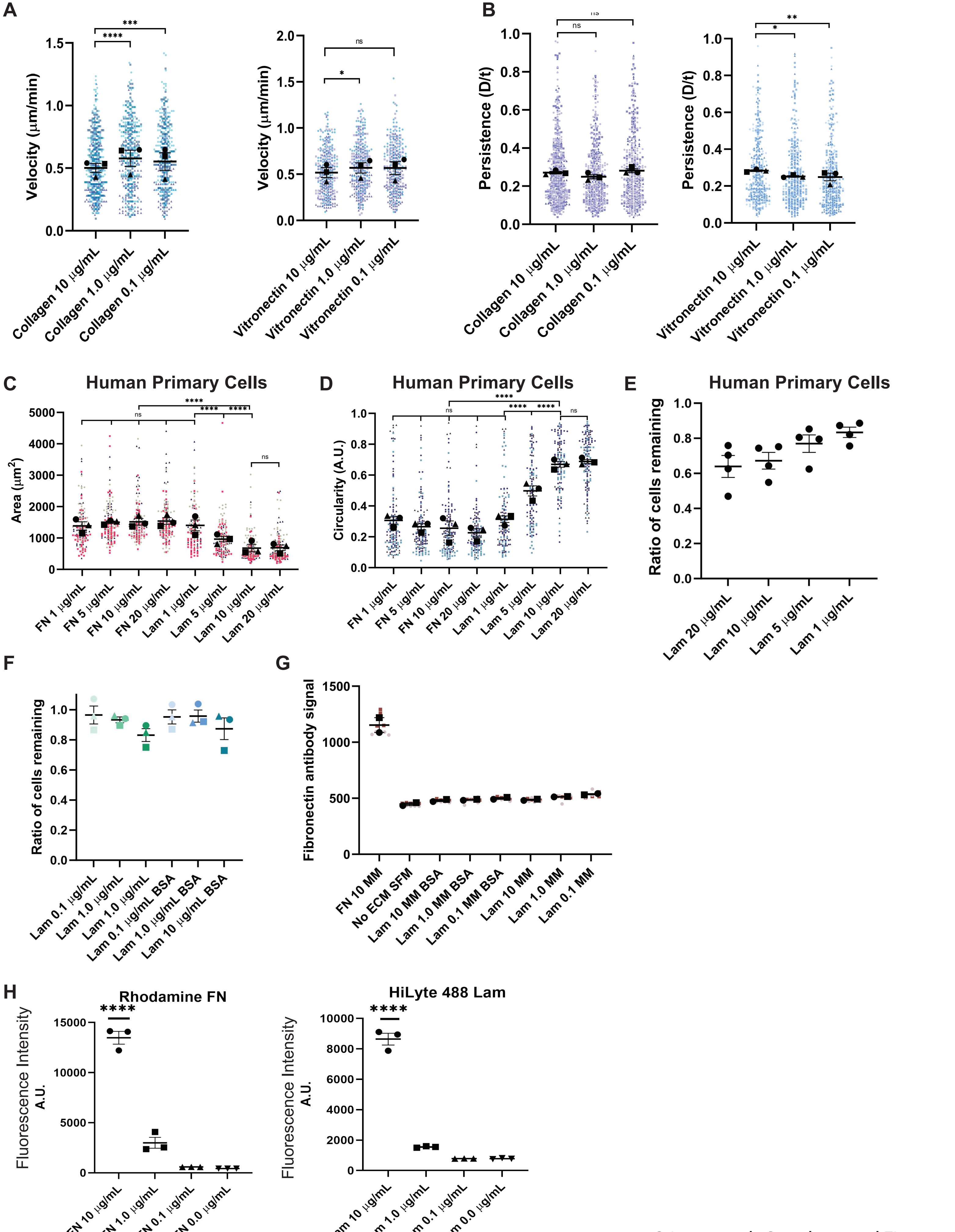

Stinson et al., Supplemental Figure 2

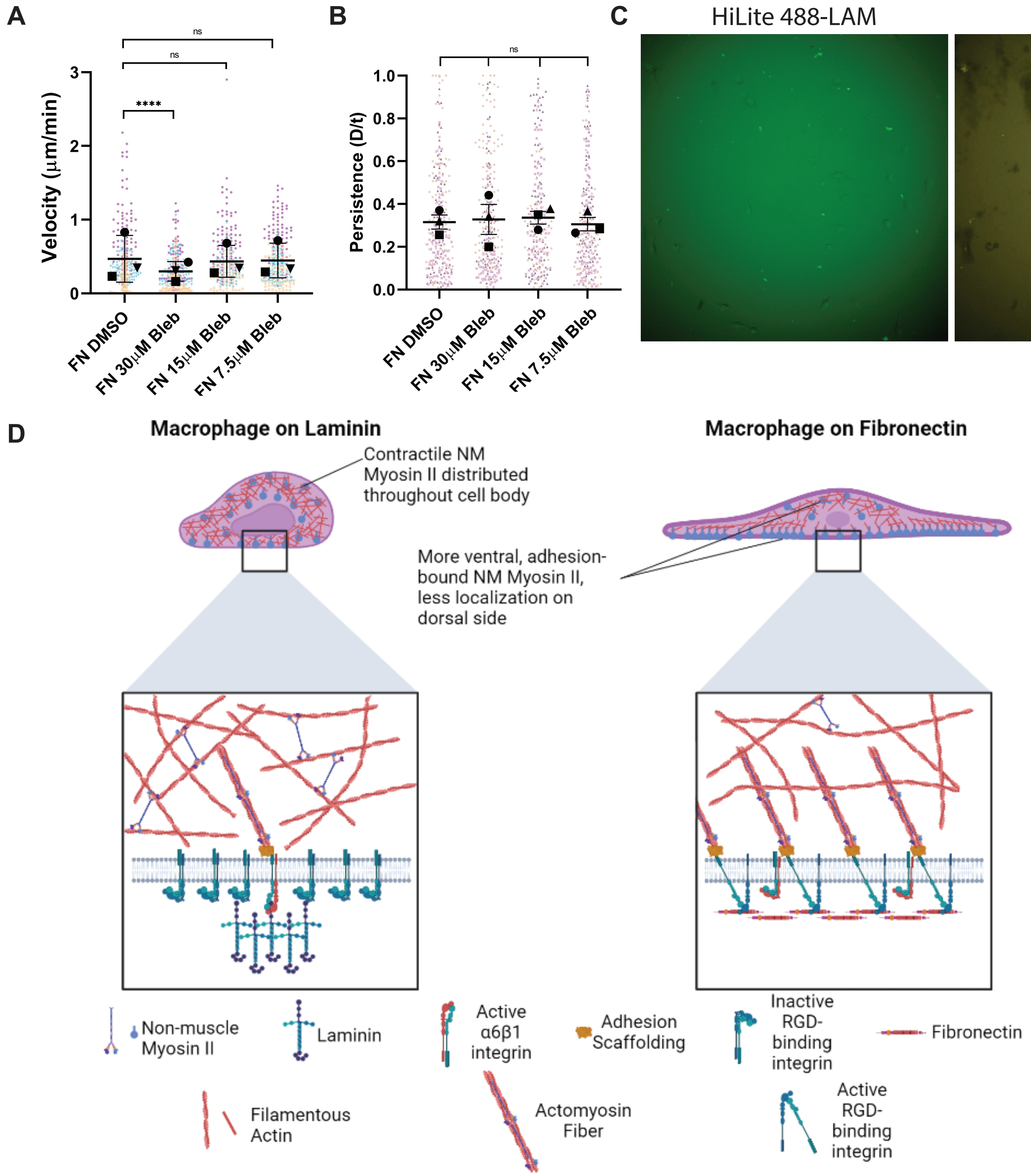

Stinson et al., Supplemental Figure 3

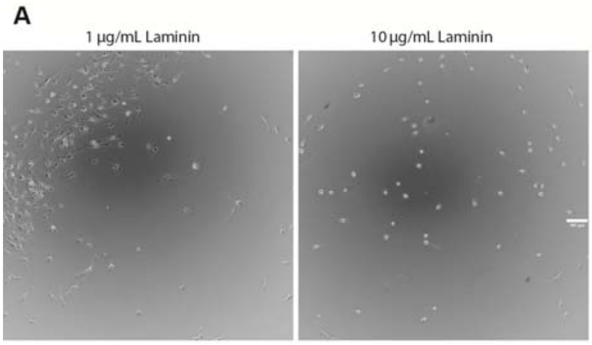

Uncropped images from Figure 2C

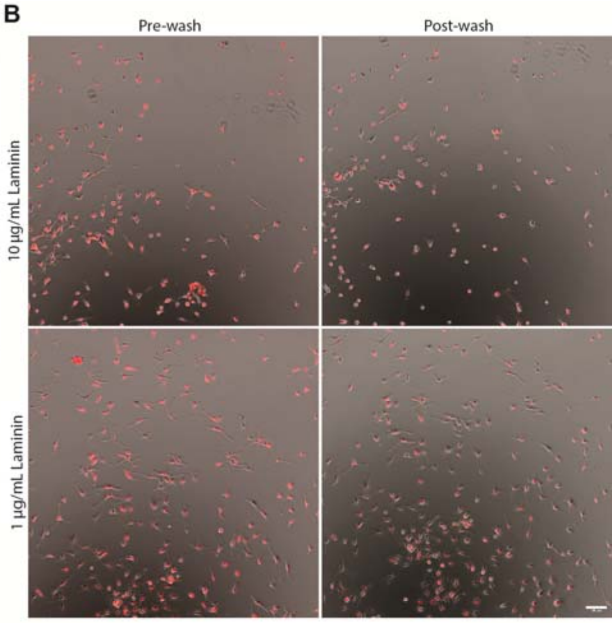

Uncropped images from Figure 2D

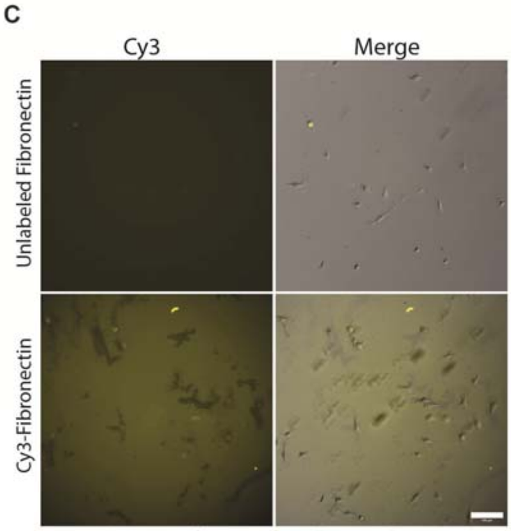

Uncropped images from Figure 6C

Stinson et al., Supplemental Figure 4

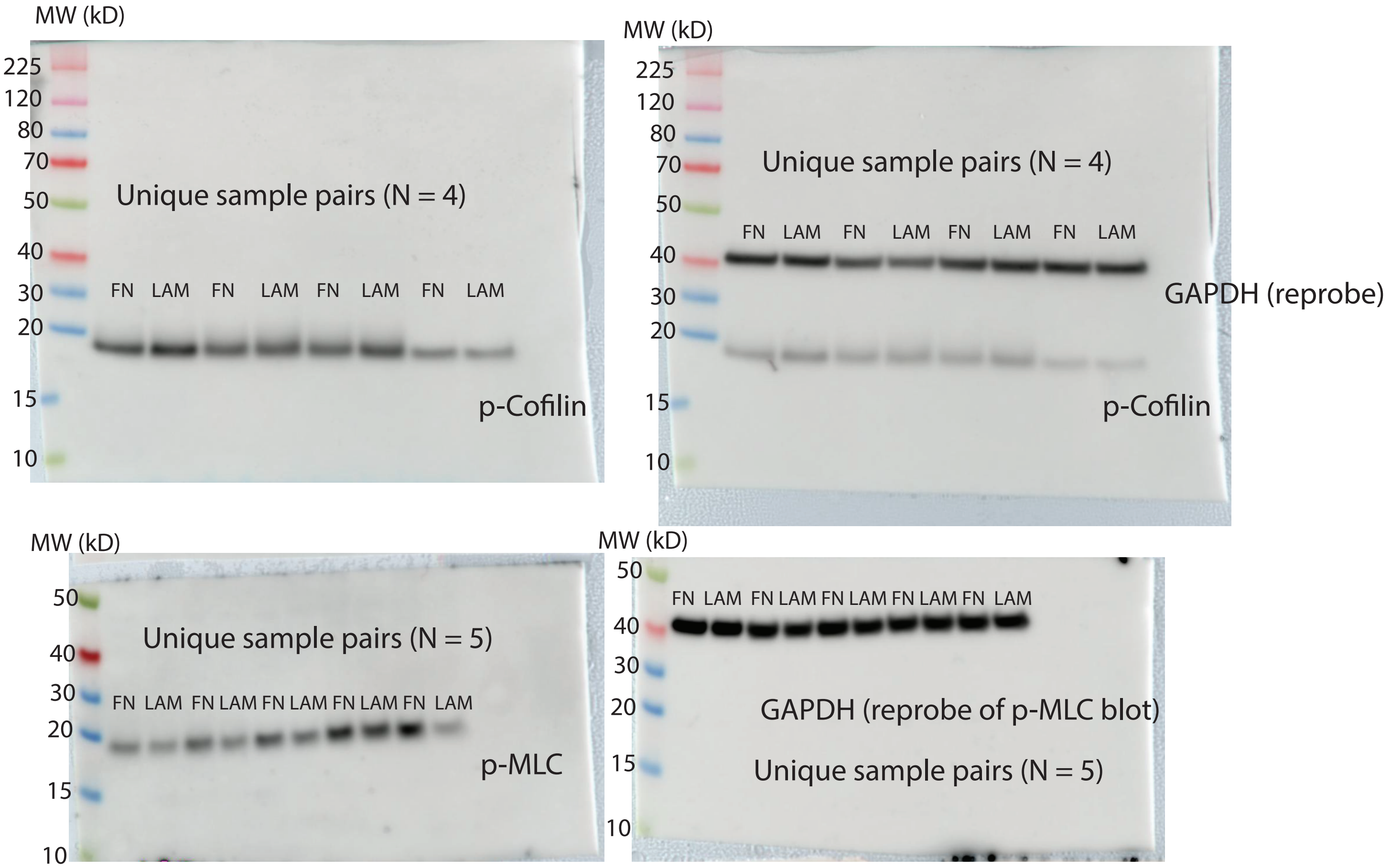

Blots from Figure 5D

Stinson et al., Supplemental Figure 5
